# Supplementary material for: More common than we thought: Hypermobile Ehlers-Danlos Syndrome, comorbidity burden, and long COVID risk in a national cohort
Source: PLOS Digit Health. 2026 Nov 5;5(11):e0001707. doi: 10.1371/journal.pdig.0001707 (PMC13739140; doi:10.1371/journal.pdig.0001707)
Supplement: Supplementary file 1 [file pdig.0001707.s011.pdf]

Critical Flaws

1. The Temporal Analysis Actually Undermines Their Conclusion

Look carefully at **Figure 3a and 3c** - their own cumulative incidence curves. The hEDS diagnoses following COVID/Long COVID show:

- Curves that rise **early (immediately) and steeply** from day 0
- Most diagnoses occur within the first 200-500 days
- The pattern is nearly identical regardless of comorbidity presence

This pattern is **classic ascertainment bias**, NOT biological unmasking.
If COVID truly "revealed" previously hidden hEDS, we would expect:

- A lag period (physiological changes take time)
- Gradual acceleration as symptoms manifest
- Differential timing based on severity or comorbidity burden

Instead, what they show is:
COVID infection → increased healthcare contact → diagnostic workup → hEDS diagnosis.
This is a surveillance artifact.

2. The "Vertebral Tuberculosis" Red Flag

In Figure 4b, they report 154 hEDS patients with "vertebral tuberculosis" versus <20 controls - an OR of 7.14.
This is **epidemiologically impossible** and reveals a fundamental coding problem:

- Vertebral TB is exceedingly rare in the US (incidence ~0.1-0.4 per 100,000)
- Finding 154 cases in 29,293 patients = ~525 per 100,000
- This is 1,000x higher than expected

What's likely happening: The ICD-10 code A18.01 (tuberculosis of spine) is likely being misused or misapplied to code chronic back pain, spinal instability, or other musculoskeletal issues in hEDS patients.
This demonstrates **severe diagnostic coding unreliability** in their dataset.

3. The Propensity Matching is Inadequate

The authors matched on demographics and "data partner" (institution) but **critically failed to match on**:

- **Healthcare utilization intensity** (number of encounters)
- **Pre-existing pain or chronic symptom diagnoses**
- **Specialist care access patterns**

hEDS patients are, by definition, high healthcare utilizers with complex symptom presentations. This creates:

- More opportunities for diagnosis
- More encounters with specialists who might recognize hEDS
- More complete diagnostic coding

4. The B94.8 Code Problem

The authors acknowledge that B94.8 ("sequelae of infectious disease") was used as a proxy for Long COVID before U09.9 existed. But look at their numbers:

- 232 hEDS patients vs 50 controls with B94.8
- OR = 4.47

This code is a **catch-all** for post-viral symptoms.
The elevated rate in hEDS patients likely reflects:

- Pre-existing chronic symptoms being retrospectively attributed to past infections
- “Diagnostic anchoring” (clinicians looking for infectious triggers in symptomatic patients)
- Coding practices in specialty clinics treating hEDS patients

5. The "Increased Paxlovid Use" Interpretation

The authors interpret higher Paxlovid prescribing rates (OR = 2.61) as evidence that hEDS patients have "moderate COVID-19 complications."
But consider:

- Paxlovid became available in late 2021, **after** awareness of Long COVID risks increased

- Clinicians may **preferentially prescribe** Paxlovid to patients with known chronic conditions (including hEDS) to prevent Long COVID – this is **provider behavior**, not disease severity

No data on hospitalization rates, ICU admission, or objective severity markers support their severity claim.

6. The Prevalence Claim

They report 1 in 800 prevalence and claim this is "higher than previously recognized" but "consistent with Demmler et al." However:

- Demmler's study (2019, Wales) used **validated clinical criteria**, not just ICD codes
- This cohort includes 27,937 "unspecified EDS" patients they **assume** are hEDS
- They offer no validation of diagnoses through chart review
- The authors explicitly acknowledge "false positives" are possible but dismiss this concern

Their prevalence estimate is likely **massively inflated** by:

- Rule-out diagnoses being coded as confirmed
- Post-2017 criteria expansion
- Diagnostic substitution during the Long COVID era

What Should Have Been Done

Rigorous analysis would include:

1. **Validation sub-study**: Chart review of a random sample to confirm hEDS diagnoses meet 2017 criteria
2. **Healthcare utilization matching**: Control for number of encounters, specialist visits, chronic pain diagnoses
3. **Temporal control**: Compare hEDS diagnosis rates over time in COVID+ vs COVID- patients, adjusted for encounter frequency
4. **Sensitivity analyses**:
    - Restrict to "diagnosed hEDS" only (exclude unspecified EDS)

- Restrict to pre-pandemic hEDS diagnoses only
- Assess for secular trends in hEDS coding independent of COVID

5. **Negative control outcomes**: Show that conditions with similar symptom overlap (fibromyalgia, chronic fatigue) don't show the same temporal pattern

Major concerns requiring revision:

1. **Title and conclusions overstate causality**: Change "Could a Viral Infection Lead to a Diagnosis?" to "Is Viral Infection Associated with Increased hEDS Diagnosis?" The current framing implies biological mechanism when the data show temporal association only.
2. **Inadequate control for ascertainment bias**: The propensity matching must include healthcare utilization intensity. Without this, any temporal association cannot be interpreted.
3. **Diagnostic validation is essential**: At minimum, a chart review sub-study of 100-200 randomly selected hEDS cases to confirm they meet 2017 criteria. The "vertebral TB" finding suggests serious coding quality issues.
4. **Figure 3 interpretation is backwards**: The early steep rise in diagnosis following COVID/LC is consistent with surveillance bias, not biological unmasking. This must be acknowledged and discussed.
5. **Secular trend analysis missing**: Show hEDS diagnosis rates over calendar time (2018-2025) stratified by COVID status. If there's a general increase in hEDS diagnosis post-2020, this undermines the specific COVID association claim.
6. **Alternative hypothesis must be seriously considered**: The discussion mentions "heightened healthcare engagement" but dismisses it. Given the data patterns, diagnostic ascertainment is the likely explanation and deserves equal weight.

This manuscript describes an interesting association but makes an unsupported causal claim. The temporal relationship they observe is entirely consistent with—and perhaps more readily explained by—increased diagnostic evaluation following COVID rather than biological unmasking of latent connective tissue disease.

The paper would be acceptable if:

- The causal language is removed throughout
- Ascertainment bias is treated as the primary alternative hypothesis
- Healthcare utilization matching is performed
- Diagnostic validation is conducted

As written, publishing this could actively harm patients by:

- Encouraging clinicians to diagnose hEDS in Long COVID patients based on symptom overlap alone
- Contributing to diagnostic inflation and unclear clinical criteria
- Potentially directing patients away from evidence-based Long COVID care toward hEDS-specific interventions without clear benefit

Recommendation: Major revision required before this can be considered for publication.
