# Supplementary material for: More common than we thought: Hypermobile Ehlers-Danlos Syndrome, comorbidity burden, and long COVID risk in a national cohort
Source: PLOS Digit Health. 2026 Nov 5;5(11):e0001707. doi: 10.1371/journal.pdig.0001707 (PMC13739140; doi:10.1371/journal.pdig.0001707)
Supplement: Supplementary file 2 [file pdig.0001707.s012.pdf]

PDIG-D-25-00524 Statistical Concerns

- No inferential testing of central claim (temporal relationship)
- Matched data analyzed as unmatched (if ORs are from 2x2 tables)
- Date-shifting uncertainty not quantified despite being fatal to temporal claims
- No sensitivity analyses
- Code not available for verification
- Competing explanations not tested statistically

Requires formal testing of temporal associations:
The central claim of viral "unmasking" requires statistical testing, not merely descriptive KM curves. Please provide log-rank tests, Cox regression with COVID-exposure as time-varying covariate, or interrupted time series analysis comparing pre/post-pandemic hEDS diagnosis rates.

Clarify OR calculation method:
For matched data, crude ORs from 2x2 tables are inappropriate. Please use conditional logistic regression or McNemar's test, or justify why matching was broken.

Address date-shifting impact:
With +/- 180 day shifts, temporal relationships within ~1 year have 72% measurement error. Please provide sensitivity analysis quantifying how this affects conclusions, or restrict analysis to events >2 years apart.

Must correct multiple comparison:
Testing 40+ conditions without correction inflates Type I error to nearly 90%. Please apply FDR correction or clearly label this as exploratory hypothesis-generation.

Code availability:
Computational reproducibility requires code deposition. Please provide complete analysis scripts in supplementary materials for public repositories.

Conclusion: The statistical analysis is technically executed, but conceptually inadequate for the claims made. The analysis describes patterns adequately but fails to test the causal hypothesis rigorously. For a PLOS Digital Health manuscript claiming to identify a biological mechanism (viral unmasking), this is insufficient. Stat methods are incomplete and inappropriate for the research question addressed.
