# Supplementary material for: More common than we thought: Hypermobile Ehlers-Danlos Syndrome, comorbidity burden, and long COVID risk in a national cohort
Source: PLOS Digit Health. 2026 Nov 5;5(11):e0001707. doi: 10.1371/journal.pdig.0001707 (PMC13739140; doi:10.1371/journal.pdig.0001707)
Supplement: Supplementary file 3 [file pdig.0001707.s013.pdf]

Referee Report for "Understanding Comorbidities in Hypermobile Ehlers-Danlos Syndrome: Could a Viral Infection Lead to a Diagnosis?"

This paper leverages a large-scale electronic health record dataset (the N3C COVID-19 Enclave) to investigate associations between hypermobile Ehlers–Danlos Syndrome (hEDS), COVID-19, and Long COVID. Given that hEDS is a complex and often under-diagnosed connective tissue disorder, the study provides valuable epidemiological insights into its prevalence, comorbidities, and post-viral outcomes. The authors' finding that individuals with hEDS face a significantly higher risk of developing Long COVID might be clinically meaningful. Moreover, the hypothesis that many hEDS diagnoses occurred after COVID-19 infection raises important questions about diagnostic delay and disease recognition. Overall, this is a clearly written and well-organized manuscript that addresses an important topic. I provide several major and minor comments below.

Major:

1. **Viral Infection and hEDS Diagnosis.** One of the key findings highlighted in the paper, including in the title, concerns the relationship between viral infection and subsequent hEDS diagnosis. This is an intriguing and potentially impactful observation.

   However, the current analysis does not appear sufficient to substantiate the claim that COVID-19 infection (or Long COVID) "acts as a physiological stressor that unmasks underlying connective tissue dysfunction."

   (i) To support the assertion that viral infection affects diagnosis rates, it would be important to present diagnostic rates both before and after infection. Comparing these rates directly would help quantify the magnitude of any effect. At present, only post-infection rates are shown, which limits the strength of the inference.

   (ii) Even if a post-infection increase in diagnosis rates were demonstrated, the proposed mechanism of "unmasking" remains speculative. Alternative explanations—such as increased healthcare contact following COVID-19 infection, which may facilitate recognition of pre-existing but undiagnosed conditions—should also be considered. The authors should discuss these competing hypotheses and clarify the rationale for favoring the physiological stressor explanation.

2. **Confidence Intervals for Prevalence Estimates.** The manuscript reports only point estimates of hEDS prevalence. It would be more informative and statistically appropriate to include confidence intervals, both for the current estimate and for

previously published ones. This would allow readers to assess whether the observed difference is statistically or substantively significant.

3. **Replication Code and Analytical Transparency.** While the N3C data cannot be directly shared, the analytic code and parameters used in the study should be made publicly available. Providing the exact analysis scripts, software versions, and parameter settings would enhance reproducibility and allow readers to better assess the robustness of the findings.

4. **Matching Procedure and Covariate Balance.** Greater transparency is needed regarding the matching procedure. The manuscript should specify the key parameters, variables, and models used to perform the matching. In addition, I recommend including a summary table (ideally in the Supplementary Materials) that presents the balance of major covariates between the hEDS and matched control groups. Such information would allow readers to assess the adequacy of the matching process and confirm that potential confounding has been appropriately controlled.

Minor:

1. **Clarification of Sample Counts.** In Figure 5, the manuscript reports 9,279 diagnosed hEDS patients and 27,937 suspected EDS patients. However, these numbers would sum to 37,216, while the text refers to a total of approximately 30,000 patients. The authors should clarify how these counts were derived—specifically, whether there is overlap between the diagnosed and suspected groups, or if different inclusion criteria or data filters were applied. A brief explanation in the Methods or Figure legend would help resolve this apparent discrepancy.

2. **Missing References.** On page 20, references [32] and [33], which appear to correspond to software used in the analysis, are missing. Please ensure that all cited tools and packages are properly referenced.
