# Supplementary material for: More common than we thought: Hypermobile Ehlers-Danlos Syndrome, comorbidity burden, and long COVID risk in a national cohort
Source: PLOS Digit Health. 2026 Nov 5;5(11):e0001707. doi: 10.1371/journal.pdig.0001707 (PMC13739140; doi:10.1371/journal.pdig.0001707)
Supplement: Supplementary file 4 [file pdig.0001707.s014.pdf]

We thank the reviewers for their very constructive feedback, which we believe has greatly strengthened the manuscript. Our detailed responses to the reviewers' comments and suggestions are highlighted in bold.

We also point out that we rearranged the sections so that the Methods section follows the Introduction similar to other recent PLOS Digital Health articles. We believe this creates a better flow for the manuscript. As a result, figure and table numbers were also reordered.

We note that the database used for the study in this manuscript has been unavailable since the end of September 2025. During this period, accessing any data, analyses, etc., was not possible. However, for 10 days in December 2025, the database was temporarily accessible to extract data and code. During this brief window, we attempted to address the reviewers' comments to the extent possible. Owing to this limited access window, not all comments could be fully addressed. We appreciate your understanding.

1. Change "Could a Viral Infection Lead to a Diagnosis?" to "Is Viral Infection Associated with Increased hEDS Diagnosis?" This is more of a hypothesis generating, not causation-proving study. It is not appropriate to conclude causation. At most can conclude association that needs further study to validate as well as to elucidate if causation or artifact. The phrase COVID-19 "acts as a physiological stressor that unmasks underlying connective tissue dysfunction." must be presented as one of several hypotheses. This type of data and analysis -- lacking clinical note validation or lab values -- are not appropriate to make conclusions about physiological processes.

- **We agree with the reviewers that the title does not adequately describe the nature of this study. However, we believe that changing it to "Could a Viral Infection Unmask the Disorder?" more accurately reflects the overall aim of the study and hypothesis at hand, emphasizing the hypothesis-generating aspect of the research. We have revised the manuscript so that any statements suggesting COVID-19 "acts as a physiological stressor that unmasks underlying connective tissue dysfunction" are clearly presented as one of several possible hypotheses. Our analysis directly examines the timing of hEDS diagnoses in relation to viral infection, but we do not draw conclusions about underlying physiological mechanisms. We hope these edits help support the idea of this unmasking event.**

2. Lack of control. This could be achieved by
a-Showing temporal trends in the years before. Compare hEDS diagnosis rates over time in COVID+ vs COVID- patients, adjusted for encounter frequency. Even so, that

would not account for healthcare utilization pattern changes in COVID. (Reviewer 2 suggestion). Secular trend analysis missing. To support the assertion that viral infection affects diagnosis rates, it would be important to present diagnostic rates both before and after infection. Comparing these rates directly would help quantify the magnitude of any effect. At present, only post-infection rates are shown, which limits the strength of the inference.

- **We apologize for not including a temporal analysis prior. We agree that presenting pre-infection diagnostic trends strengthens the interpretation of our findings. To address this, we have added Figure 4, which shows hEDS diagnostic status from 2018 to the present, segmented by COVID-19 infection (4a) and long COVID development (4b). Dotted lines represent raw counts, while solid lines are normalized per 1,000 encounters. Additionally, Supplemental Figure 2 shows an interrupted time-series analysis of monthly hEDS diagnoses from pre-pandemic through post-pandemic periods. Table 3 further quantifies diagnostic timing relative to COVID-19 infection, offering a clearer view of potential temporal associations.**

b-Comparison to a comparable condition, fibromyalgia, or chronic pain. (Reviewer 3 suggestion) The analysis needs to extend to both of these to better address confounding or bias

Also: The Propensity Matching is Inadequate. The authors matched on demographics and "data partner" (institution) but critically failed to match on: Healthcare utilization intensity (number of encounters), Pre-existing pain or chronic symptom diagnoses, Specialist care access patterns. hEDS patients are, by definition, high healthcare utilizers with complex symptom presentations. This creates: More opportunities for diagnosis, More encounters with specialists who might recognize hEDS, More complete diagnostic coding

- **We agree that appropriate controls are critical. We re-performed propensity matching to include total healthcare utilization (macrovisits combined across emergency department, inpatient, and outpatient visits per day). Specialty care access patterns were too sparsely reported in the database for matching.**
- **We respectfully note that using conditions such as fibromyalgia or chronic pain as comparators could create bias, as these often co-occur with hEDS and many patients with these conditions eventually receive an hEDS diagnosis, leading to false positives. We looked at other connective tissue disorders (e.g., Loeys-Dietz, Marfan syndrome), but these groups were too small for meaningful comparison. We acknowledged this limitation in the manuscript and emphasized the need for follow-up studies with broader**

comparator cohorts. This is highlighted in the Limitations section (Paragraph 4).

3. Provide a summary table (ideally in the Supplementary Materials) that presents the balance of major covariates between the hEDS and matched control groups.
a. Also include a new variable of site of visit or coding (specialist, ED, primary care, etc) as coding practices will differ by location and clinician type

- **We thank the reviewers for this suggestion. We agree that presenting covariate balance is important. Accordingly, we have added a summary table in the Supplementary Materials (Supplementary Table 3: Covariates of Cases and Controls). This table includes age, sex, race, ethnicity, data partner, type of visit (ED, inpatient, outpatient), and total visits. Categorical variables are reported as raw counts, and continuous variables are reported as means with standard deviations. Including the type of visit helps account for potential differences in coding practices across sites and clinician types, providing a clearer context for interpreting the matched cohorts.**

4. Clearly address the issue of false positives and false negatives via wrong coding. The study design of Demmler et all (2019, Wales) used validated clinical criteria, not just ICD codes. This needs to be addressed more clearly in the discussion with clarity that this is methodologically very different from Demmler et al. The discussion should call for clinical validation as future research.

Also: The Prevalence Claim. They report 1 in 800 prevalence and claim this is "higher than previously recognized" but "consistent with Demmler et al." However: Demmler's study (2019, Wales) used validated clinical criteria, not just ICD codes, This cohort includes 27,937 "unspecified EDS" patients they assume are hEDS, They offer no validation of diagnoses through chart review, The authors explicitly acknowledge "false positives" are possible but dismiss this concern. Their prevalence estimate is likely massively inflated by: Rule-out diagnoses being coded as confirmed, Post-2017 criteria expansion, Diagnostic substitution during the Long COVID era

a. One way to address this and verify: a chart review sub-study of 100-200 randomly selected hEDS cases to ensure they meet diagnostic criteria

- **We recognize that relying on EHR diagnosis codes may lead to misclassification. Manual chart review was not allowed within N3C. Key diagnostic features for hEDS, such as the Beighton Score or multisystem features like “unusually soft or velvety skin” are not captured by ICD or LOINC codes, which prevents clinical validation. We have clarified this limitation in the Discussion: “Clinical validation through chart review was not possible, limiting interpretation; false positives or misclassifications**

could occur due to rule-out diagnoses being coded as confirmed, post-2017 criteria expansion, or diagnostic substitution during the Long Covid era. Future research should include chart-based validation to confirm these findings.”

- **We respectfully note that our approach is consistent with Demmler et al.** [1], **who also used ICD-10 and Read Codes instead of fully validated clinical criteria. We have added a discussion of this methodological limitation and the potential for misclassification bias it introduces in the limitation section in Discussion.**

b. address the issue of B94.8 ("sequelae of infectious disease") was used as a proxy for Long COVID before U09.9 existed -- this can be addressed by extending analysis to pre-COVID years to show what the baseline was

- **Given the very limited time we had with the data during the short 10-day window when N3C was back online in December, we were not able to address this directly. Instead, we have noted it as a limitation in the discussion. “ However, this code was also often applied to pre-existing post-viral symptoms, highlighting limitations of code-based inference. Further follow-up of the timing of this diagnostic code is necessary to reach a definitive conclusion.”**

c. address " 154 hEDS patients with "vertebral tuberculosis" versus <20 controls - an OR of 7.14. This is epidemiologically impossible and reveals a fundamental coding problem: Vertebral TB is exceedingly rare in the US (incidence ~0.1-0.4 per 100,000)" -- how reliable are these data and what is evidence of mislabeling or coding error?

- **We thank the reviewers for highlighting this issue. Upon investigation, the reported link between hEDS and “vertebral tuberculosis” could not be reliably explained. These diagnosis codes came from multiple clinical sites, and no consistent pattern emerged that could account for this unexpected finding. Given the extreme rarity of vertebral tuberculosis in the U.S. (incidence ~0.1 - 0.4 per 100,000), and the absence of supporting clinical context, we suspect these codes may reflect misclassification, possibly where lower back pain or other spinal conditions were mistakenly coded as vertebral TB. Because we cannot confidently interpret these data, we have removed this analysis from the manuscript and noted this as a limitation in the Discussion.**

5. Clarification of Sample Counts. In Figure 5, the manuscript reports 9,279 diagnosed hEDS patients and 27,937 suspected EDS patients. However, these numbers would sum to 37,216, while the text refers to a total of approximately 30,000

patients. The authors should clarify how these counts were derived—specifically, whether there is overlap between the diagnosed and suspected groups, or if different inclusion criteria or data filters were applied.

- **We thank the reviewers for pointing out the need for clarification. After re-analyzing the propensity matching to include healthcare utilization, we reconstructed the cohort. Between the initial data freeze (April 2025) and the December 2025 freeze, some clinical sites removed patient information from N3C, resulting in decreases in the overall counts. To clarify the sample sizes, we have updated both the figure legend and the text. Specifically, 5,709 patients had both unspecified EDS and hEDS coded in their charts, and only 216 patients had diagnoses of cEDS or vEDS along with an unspecified EDS diagnosis; these overlapping cases were removed from the analyses. These updates provide a clear description of cohort construction and explain the differences in reported numbers.**

6. Missing References. On page 20, references [32] and [33], which appear to corresponds to software used in the analysis, and are missing. Please ensure that all cited tools and packages are properly referenced.

- **We thank the reviewers for noting this oversight. References [32] and [33] have been added to properly cite the software and packages used in the analysis.**

7. hEDS prevalence:It would be more informative and statistically appropriate to include confidence intervals, both for the current estimate and for previously published ones. This would allow readers to assess whether the observed difference is statistically or substantively significant.

Also: Prevalence Estimation Biases: The reported higher prevalence of hEDS (~1 in 800) in N3C could be due to selection bias, the N3C cohort overrepresents individuals with greater healthcare utilization such as hEDS.

- **We thank the reviewers for the suggestion. Confidence intervals for the estimated hEDS prevalence in N3C have been added to Table 1 and described in the Results section under "hEDS is more prevalent than previously recorded." Previously published prevalence estimates did not include confidence intervals, so we did not report them.**
- **Regarding potential bias, while we acknowledge that N3C includes patients with varying healthcare utilization, the network comprises a diverse mix of clinical sites, from small clinics to large hospitals, providing a broad representation. Therefore, we believe the observed prevalence (~1 in 800)**

reflects the most comprehensive estimate of hEDS in the U.S. available to date.

8. Mislabeling of related conditions, especially dysautonomia, POTS, and MCAS as hEDS is a very common practice. Therefore, considering nonspecific/General EDS as hEDS is highly plausible, specifically during the COVID-19 pandemic and overwhelmed healthcare systems. In addition, information bias caused by inconsistent documentation and variable use of diagnostic codes across institutions further degrade the diagnostic fidelity of hEDS within registry data. These biases should be considered in the limitation section.

- **We agree that misclassification and information bias are important considerations when studying hEDS using registry-based data. Conditions such as dysautonomia, POTS, MCAS, and nonspecific EDS may be variably documented as hEDS, particularly during periods of healthcare strain such as the COVID-19 pandemic, and diagnostic coding practices vary across institutions. We have now explicitly addressed these limitations in the limitation section of the manuscript. Our analyses aim to evaluate temporal and comparative patterns of hEDS-coded diagnoses rather than establish definitive clinical diagnoses. While nonspecific or imprecise use of hEDS-related codes could inflate post-COVID diagnoses, we observe structured, condition-specific temporal trends and consistent differences across comparator groups, suggesting that documentation artifacts alone do not explain our findings. Where feasible, we excluded nonspecific EDS codes to improve diagnostic accuracy, though residual misclassification remains a limitation.**

9. The inclusion of phenotypes such as infertility, autism spectrum disorder, structural gastrointestinal anomalies, and movement disorders as hEDS-related phenotypes lacks supportive evidence. While such associations may be worthy of hypothesis generation, their presentation as established findings is premature and potentially misleading.

- **We thank the reviewers for this comment. In response, we replaced infertility with pre-eclampsia, postpartum bleeding, and hyperemesis gravidarum based on findings from a childbearing hEDS cohort** [2] **(Pearce et al., 2023), as shown in Figure 1. However, we respectfully request that certain other phenotypes be kept as relevant to hEDS due to supporting evidence. Autism spectrum disorder (ASD) has been shown to be highly correlated with hEDS** [3, 4]**(Baeza-Velasco et al., 2025; Cederlof et al., 2016). Structural gastrointestinal anomalies are supported by multiple studies** [5, 6]**(Fikree et al., 2017; Aziz et al., 2025) and encompass conditions such as hiatus hernia, visceroptosis, rectocele, rectal prolapse, and abdominal wall**

hernias. Movement disorders, including tremors, dystonia, gait and balance issues, and functional movement disorders, are also consistently observed in hEDS cohorts [7, 8]**(Margolesky et al., 2022; Fernandez et al., 2024). We present these phenotypes as hypothesis-generating but supported by literature, rather than definitive causal links, to highlight potentially important features of hEDS.**

11. Replication Code and Analytical Transparency. While the N3C data cannot be directly shared, the analytic code and parameters used in the study should be made publicly available. Providing the exact analysis scripts, software versions, and parameter settings would enhance reproducibility and allow readers to better assess the robustness of the findings.

- **We thank the reviewers for this suggestion. To improve reproducibility, we have made all analytic code, software versions, and parameter settings publicly available via a GitHub repository. A Data and Code Availability statement has been added to the manuscript, and the Methods section has been revised to reference this repository. Additionally, a Code Availability and Reproducibility in the Supplementary has been added to the manuscript.**

11. Several explicit claims in the discussion fall outside the scope of the data presented. For example: “The elevated odds of tuberculosis, for instance, may reflect increased susceptibility due to immune dysregulation or preexisting lung pathology in hEDS patients.” “The increased use of systemic corticosteroids is also notable, given their known adverse effects on connective tissue integrity.” or “Finally, the elevated odds of hemiplegia or paraplegia in the hEDS population may reflect neurological complications stemming from central nervous system fragility or spinal instability, which are increasingly recognized as potential hEDS manifestations.”

- **We thank the reviewers for highlighting these statements. We agree that causal conclusions cannot be drawn from our data. These passages in the Discussion are intended to provide potential, hypothesis-generating explanations for the observed associations and are explicitly speculative. We have revised the language to clarify this framing by using qualifiers such as “may” or “could reflect” to indicate that these interpretations are not established mechanisms. The goal of this section is to contextualize the findings and suggest plausible biological or clinical explanations, while emphasizing that further research, including clinical validation and mechanistic studies, is required to evaluate these hypotheses.**

12. Even if a post-infection increase in diagnosis rates were demonstrated, the proposed mechanism of “unmasking” remains speculative. Alternative explanations—such as increased healthcare contact following COVID-19 infection, which may facilitate recognition of pre-existing but undiagnosed conditions—should also be considered. The authors should discuss these competing hypotheses and clarify the rationale for favoring the physiological stressor explanation.

Also: Alternative hypothesis must be seriously considered: The discussion mentions "heightened healthcare engagement" but dismisses it. Given the data patterns, diagnostic ascertainment is the likely explanation and deserves equal weight.

- **We thank the reviewers for highlighting this important point. We agree that the “unmasking” mechanism is speculative and that alternative explanations, including increased healthcare utilization after COVID-19 infection, might contribute to higher diagnosis rates of hEDS. We have revised the Discussion to explicitly present these competing hypotheses with equal emphasis. While our data are consistent with a potential physiological stressor effect, we clarify that increased healthcare contact, diagnostic ascertainment, and other systemic factors are equally plausible explanations. Our interpretation now highlights that these findings are hypothesis-generating, and further studies, including clinical validation and mechanistic research, are necessary to disentangle these possibilities.**

13. Requires formal testing of temporal associations: The central claim of viral “unmasking” requires statistical testing, not merely descriptive KM curves. Please provide log-rank tests, Cox regression with COVID-exposure as time- varying covariate, or interrupted time series analysis comparing pre/post-pandemic hEDS diagnosis rates.

- **We thank the reviewers for highlighting the need for formal testing of temporal associations. To address this, we performed several analyses to quantify changes in hEDS diagnosis rates related to COVID-19 exposure. First, we conducted Kaplan-Meier cumulative incidence analyses and log-rank tests comparing the timing of hEDS diagnosis between patients with and without COVID-19 or Long COVID. To account for time-dependent exposure, we also implemented Cox proportional hazards models with COVID-19 diagnosis modeled as a time-varying covariate. Finally, we performed an interrupted time-series analysis using monthly encounter-adjusted hEDS diagnosis rates from 2018 through 2023, enabling us to compare trends before, during and after the pandemic. These analyses consistently support the temporal link between COVID-19 exposure and subsequent hEDS diagnosis, while adjusting for differential follow-up and encounter frequency. The methods, results, and figures for**

these analyses are described in the Results section (Figures 4 and Supplemental Figure 2) and corresponding tables (Table 3).

14. Clarify OR calculation method: For matched data, crude ORs from 2x2 tables are inappropriate. Please use conditional logistic regression or McNemar's test, or justify why matching was broken.

- **We thank the reviewers for raising this point. To account for the matched study design, we calculated odds ratios using conditional logistic regression, which preserves the matching structure and adjusts for within-pair correlation. The Methods section has been updated to specify the use of conditional logistic regression for all matched analyses, and the corresponding confidence intervals and p-values now reflect this approach.**

15. Address date-shifting impact: With +/- 180 day shifts, temporal relationships within ~1 year have 72% measurement error. Please provide sensitivity analysis quantifying how this affects conclusions, or restrict analysis to events >2 years apart.

- **We thank the reviewers for noting the potential impact of date-shifting. Because our Kaplan-Meier analyses and cumulative incidence estimates are based on individual-level data, the +/-180 day date shifts do not change the relative ordering of events within each individual. Similarly, the key temporal relationships between COVID-19 diagnosis and subsequent hEDS diagnosis are preserved, as our analyses are based on time from COVID-19 exposure to hEDS diagnosis rather than absolute calendar dates. We have added a statement in the Limitations section noting that date-shifting could affect fine-grained analyses of short-term trends but does not meaningfully alter the conclusions about temporal associations at the individual level.**

16. Must correct multiple comparison: Testing 40+ conditions without correction inflates Type I error to nearly 90%. Please apply FDR correction or clearly label this as exploratory hypothesis-generation.

- **We thank the reviewers for noting the potential for inflated Type I error due to multiple comparisons. We have implemented a false discovery rate (FDR) correction to all analyses involving multiple condition comparisons. Corrected q-values are now reported alongside odds ratios and confidence intervals in the Results tables and figures. In the text, we explicitly indicate which associations remain statistically significant after FDR adjustment. We also note that, despite correction, these analyses are primarily**

exploratory and hypothesis-generating, and findings should be interpreted with caution until validated in independent datasets.

17. The Temporal Analysis Actually Undermines Their Conclusion. Look carefully at Figure 3a and 3c - their own cumulative incidence curves. The hEDS diagnoses following COVID/Long COVID show: Curves that rise early (immediately) and steeply from day 0, Most diagnoses occur within the first 200-500 days, The pattern is nearly identical regardless of comorbidity presence. This pattern is classic ascertainment bias, NOT biological unmasking. If COVID truly "revealed" previously hidden hEDS, we would expect: A lag period (physiological changes take time), Gradual acceleration as symptoms manifest, Differential timing based on severity or comorbidity burden. Instead, what they show is: COVID infection → increased healthcare contact → diagnostic workup → hEDS diagnosis. This is a surveillance artifact.

- **We agree that the early and steep rise in hEDS diagnoses after COVID-19 or Long COVID could be due to increased healthcare contact and diagnostic efforts, consistent with classic ascertainment or surveillance bias. However, we note several points: first, while raw cumulative incidence curves show early rises, encounter-adjusted diagnosis rates remain stable over time, suggesting that the temporal patterns are not solely driven by healthcare utilization. Second, the increased incidence is more pronounced among patients with specific comorbidities, such as POTS or MCAS, supporting the idea that symptom clusters may trigger additional diagnostic evaluation. We have revised the Discussion to explicitly present increased healthcare engagement as a competing explanation alongside the "unmasking" hypothesis. We now frame post-COVID hEDS diagnoses as likely reflecting a combination of increased clinical attention, phenotypic clustering, and possible symptom worsening , without claiming direct biological causation. This highlights that the observed temporal link is hypothesis-generating and needs further validation through chart review or mechanistic studies.**

18. The "Increased Paxlovid Use" Interpretation. The authors interpret higher Paxlovid prescribing rates (OR = 2.61) as evidence that hEDS patients have "moderate COVID-19 complications." But consider: Paxlovid became available in late 2021, after awareness of Long COVID risks Increased, Clinicians may preferentially prescribe Paxlovid to patients with known chronic conditions (including hEDS) to prevent Long COVID – this is provider behavior, not disease severity, No data on hospitalization rates, ICU admission, or objective severity markers support their severity claim.

- **We have revised the Discussion to clarify that the observed higher Paxlovid prescribing rates among patients with hEDS may reflect provider behavior rather than disease severity. Specifically, Paxlovid became available in late 2021, coinciding with increased awareness of Long COVID risks, and clinicians may have preferentially prescribed it to patients with known chronic conditions such as hEDS as a preventative measure. We called for more research on hospitalization, ICU admission, or objective markers of COVID-19 severity to support these claims. We have also added language emphasizing that these findings should be interpreted cautiously and do not imply causal differences in COVID-19 severity.**

Citations:

1. Demmler JC, Atkinson MD, Reinhold EJ, Choy E, Lyons RA, Brophy ST. Diagnosed prevalence of Ehlers-Danlos syndrome and hypermobility spectrum disorder in Wales, UK: a national electronic cohort study and case-control comparison. BMJ Open. 2019;9: e031365.

2. Pearce G, Bell L, Pezaro S, Reinhold E. Childbearing with hypermobile Ehlers-Danlos syndrome and Hypermobility Spectrum Disorders: A large international survey of outcomes and complications. Int J Environ Res Public Health. 2023;20: 6957.

3. Baeza-Velasco C, Vergne J, Poli M, Kalisch L, Calati R. Autism in the context of joint hypermobility, hypermobility spectrum disorders, and Ehlers-Danlos syndromes: A systematic review and prevalence meta-analyses. Autism. 2025;29: 1939–1958.

4. Cederlöf M, Larsson H, Lichtenstein P, Almqvist C, Serlachius E, Ludvigsson JF. Nationwide population-based cohort study of psychiatric disorders in individuals with Ehlers-Danlos syndrome or hypermobility syndrome and their siblings. BMC Psychiatry. 2016;16: 207.

5. Fikree A, Chelimsky G, Collins H, Kovacic K, Aziz Q. Gastrointestinal involvement in the Ehlers-Danlos syndromes. Am J Med Genet C Semin Med Genet. 2017;175: 181–187.

6. Aziz Q, Harris LA, Goodman BP, Simrén M, Shin A. AGA clinical practice update on GI manifestations and autonomic or immune dysfunction in hypermobile Ehlers-danlos syndrome: Expert review. Clin Gastroenterol Hepatol. 2025;23: 1291–1302.

7. Margolesky J, Puentes D, Espay AJ. Hypermobile Ehlers-Danlos syndrome: A prodromal subtype of functional movement disorders? Mov Disord Clin Pract.

2022;9: 956–960.

8. Fernandez A, Jaquet M, Aubry-Rozier B, Suter M, Aybek S, Berna C. Functional neurological signs in hypermobile Ehlers-Danlos syndrome and hypermobile spectrum disorders with suspected neuropathic pain. Brain Behav. 2024;14: e3441.
