# Supplementary material for: More common than we thought: Hypermobile Ehlers-Danlos Syndrome, comorbidity burden, and long COVID risk in a national cohort
Source: PLOS Digit Health. 2026 Nov 5;5(11):e0001707. doi: 10.1371/journal.pdig.0001707 (PMC13739140; doi:10.1371/journal.pdig.0001707)
Supplement: Supplementary file 5 [file pdig.0001707.s015.pdf]

Referee Report for "Understanding Comorbidities in Hypermobile Ehlers-Danlos Syndrome: Could a Viral Infection Unmask the Disorder?"

I appreciate the authors' efforts to address the previous round of review by adding more comprehensive analyses. These additional analyses provide important information regarding the central hypothesis of the manuscript. However, after reviewing the revised results, I remain concerned that the evidence does not sufficiently support the manuscript's main interpretation. In particular, some of the newly added analyses appear to weaken, rather than strengthen, the argument that viral infection may unmask hEDS. My main concerns are outlined below.

1. **Comparability of the matched cohort**

   A central issue is whether the matched control cohort provides an appropriate comparison group for the hEDS cohort. In Figure 3 of the revised manuscript, the conditional odds ratio for patient death is reported as 0.43, with a relatively narrow confidence interval, comparing the hEDS cohort with the control cohort. This is a substantial difference and, in my view, requires careful explanation.

   Similar concerns apply to other outcomes with statistically significant odds ratios, such as pneumonia (OR = 0.66) and positive PCR test (OR = 0.69). These differences raise the possibility that the matched control cohort may not be sufficiently comparable to the hEDS cohort with respect to important observed or unobserved factors. For example, the hEDS cohort may differ from the control cohort in baseline health status, health-care access, care-seeking behavior, testing behavior, or surveillance intensity. If so, the observed associations may be strongly affected by residual or unmeasured confounding.

   This concern is particularly important because the paper's conclusions rely on interpreting these associations as informative for hypothesis generation. If the matched cohorts differ substantially in mortality, pneumonia, and testing-related outcomes, then the basis for drawing biological or etiologic hypotheses becomes less secure. I encourage the authors to directly address this issue and clarify what these differences imply about the validity of the matched comparison.

   In addition, the manuscript states, when summarizing Figure 3, that "testing-related variables, such as the presence of a negative PCR or antibody test, showed no difference between groups, nor did outcomes like pneumonia or mortality following COVID-19 infection." This statement does not appear to be consistent with the revised Figure 3, where several of these outcomes do show differences. The authors should revise this text to accurately reflect the updated analysis results and discuss their implications.

2. **Interpretation of temporal trends in first hEDS diagnoses relative to COVID-19**

   Figure 4 presents encounter-adjusted diagnosis rates for hEDS. This is a useful addition to the manuscript because it directly addresses a key concern: whether apparent increases in hEDS diagnoses could be driven by differential health-care utilization or surveillance.

However, my reading of Figure 4 is that, after accounting for the number of encounters, there is little evidence of a clear temporal increase in hEDS diagnosis rates after 2020. In addition, the encounter-adjusted diagnosis rates appear broadly similar across the three groups: patients diagnosed with COVID before hEDS diagnosis, patients diagnosed with COVID after hEDS diagnosis, and patients without COVID diagnosis.

This result seems difficult to reconcile with the manuscript's central framing that viral infection may unmask hEDS. An alternative interpretation is that the apparent association between COVID-related variables and hEDS diagnosis may be largely explained by health-care seeking, encounter frequency, or surveillance processes. If the authors disagree with this interpretation, they should explain more clearly what feature of Figure 4 supports the proposed hypothesis.

More generally, I think the authors should be cautious in using these results to support a viral-unmasking hypothesis. The current evidence appears more consistent with a descriptive association between hEDS, COVID-related diagnoses, and patterns of health-care utilization than with evidence that viral infection contributes to the recognition or onset of hEDS.

3. **Overall assessment and suggested reframing**

   In summary, I appreciate the additional analyses included in the revision. Nevertheless, the revised results raise substantial concerns about the interpretation of the study. The differences observed in Figure 3 suggest possible non-comparability of the matched cohorts, and the encounter-adjusted temporal trends in Figure 4 do not appear to provide strong support for the manuscript's central hypothesis.

   In my view, the manuscript would require substantial reframing before the conclusions could be considered adequately supported by the data. A more cautious framing as a descriptive study of comorbidity patterns, diagnostic timing, and health-care utilization among patients with hEDS may be more appropriate. The title, abstract, discussion, and conclusion should be revised accordingly to avoid implying stronger evidence for a viral-unmasking mechanism than the current analyses can support.

   Without such reframing, or without additional analyses that more convincingly address residual confounding, differential surveillance, and cohort comparability, I do not think the current evidence provides sufficient support for the manuscript's main claims.
