# Supplementary material for: More common than we thought: Hypermobile Ehlers-Danlos Syndrome, comorbidity burden, and long COVID risk in a national cohort
Source: PLOS Digit Health. 2026 Nov 5;5(11):e0001707. doi: 10.1371/journal.pdig.0001707 (PMC13739140; doi:10.1371/journal.pdig.0001707)
Supplement: Supplementary file 6 [file pdig.0001707.s016.pdf]

We thank the editor and reviewers for their careful reading of the revised manuscript. Below we address each comment in turn. Our detailed responses to the reviewers' comments and suggestions are highlighted in bold.

1. Revise the title so that it describes an association with increased hEDS diagnosis rather than implying biological unmasking, and align the abstract, author summary, discussion, and conclusions with this framing.

We have revised the title to "*Hypermobile Ehlers-Danlos Syndrome Is Diagnosed More Often After COVID-19: Prevalence, Comorbidities, and Diagnostic Timing*" to reflect the descriptive nature of the study.

Additionally, we have updated the abstract, author summary, discussion, and conclusion to include these changes as well.

2. Give the surveillance and diagnostic-ascertainment interpretation genuinely equal weight throughout, and reconsider the interpretation of Figure 4 so that the encounter-adjusted findings are not presented as evidence for unmasking when they are at least equally consistent with an ascertainment account.

We agree that the manuscript should not privilege one mechanistic interpretation over the other. We have revised the abstract, author summary, Figure 4 results paragraph, discussion, and conclusion to present biological unmasking and increased post-viral healthcare utilization as equally plausible, mutually non-exclusive explanations.

3. State explicitly, in the discussion and limitations, that in the absence of a comparator condition and chart-level validation the data cannot distinguish between the competing explanations, and clearly position the work as descriptive and hypothesis-generating.

We have added the following sentence to the end of the ascertainment paragraph in the Discussion: "Chart-level validation of hEDS diagnoses and a comparator cohort unrelated to connective tissue pathology would be required to formally separate these explanations; in their absence, these findings are best understood as descriptive and hypothesis-generating."

We have also added a corresponding sentence to the Limitations: "Without such a comparator, it is not possible to determine whether the observed temporal patterns in hEDS diagnosis are specific to connective tissue pathology or reflect broader trends in chronic condition recognition following COVID-19."

4. Address the consistency point raised regarding Figure 3, where the text summarising testing-related and outcome variables should be reconciled with the differences now visible in the revised figure.

We thank the reviewer for flagging these issues. The prior text was an error carried over from an earlier analysis version and did not reflect the updated

results. We have corrected the results paragraph to accurately describe all significant ORs, including those below 1.0 "In contrast, patients with hEDS had significantly lower odds of Remdesivir use (OR = 0.63, FDR = 3.2e-6), positive PCR test (OR = 0.69, FDR < 1e-10), positive antibody test (OR = 0.69, FDR < 1e-10), pneumonia following COVID-19 (OR = 0.66, FDR = 2.1e-6), and death (OR = 0.43, FDR < 1e-10). These lower odds may reflect differences in testing behavior, earlier outpatient antiviral treatment, and residual differences in baseline health status or healthcare access between the matched cohorts, and should be interpreted in that context."

We appreciate the reviewer's concern and agree that these findings require careful interpretation. We note that the propensity score matching specifically included healthcare utilization (total visit counts, stratified by visit type) as a matching covariate, alongside age, sex, race, and clinical site, specifically to reduce confounding by care-seeking behavior. The intent was to construct a control group with similar encounter frequency, so that differences in outcomes reflect something beyond pure healthcare access.

Within that context, the pattern of lower ORs for Remdesivir, pneumonia, and death alongside substantially higher odds of Paxlovid use (OR = 2.05, 95% CI 1.89–2.21) is internally consistent: it suggests that patients with hEDS disproportionately received early outpatient antiviral therapy, potentially preventing progression to inpatient settings where Remdesivir is administered and where pneumonia and death are more likely to be recorded. We acknowledge, however, that this interpretation is speculative, and that residual confounding by baseline health status, care-seeking behavior, and testing practices cannot be excluded. We have added language in the Discussion to address this directly:

"Together, these patterns suggest that patients with hEDS may be more likely to receive early outpatient treatment, potentially preventing disease progression to hospitalization, though residual differences in baseline health status and healthcare access between the matched cohorts could also contribute to these findings."

We agree with the reviewer that these differences limit the certainty with which biological or etiologic conclusions can be drawn from the matched comparison, and we have revised the manuscript throughout to frame all findings as descriptive and hypothesis-generating rather than mechanistically explanatory.
