# Supplementary material for: More common than we thought: Hypermobile Ehlers-Danlos Syndrome, comorbidity burden, and long COVID risk in a national cohort
Source: PLOS Digit Health. 2026 Nov 5;5(11):e0001707. doi: 10.1371/journal.pdig.0001707 (PMC13739140; doi:10.1371/journal.pdig.0001707)
Supplement: Supplementary file 7 [file pdig.0001707.s001.docx]

**S1 Table: Concept IDs for Cohort Criteria**

| Label | Type of Concept ID | OMOP ID |
| --- | --- | --- |
| Hypermobile Ehlers-Danlos Syndrome | Condition | 4148925 |
| General Ehlers-Danlos Syndrome | Condition | 79145 |
| Vascular Ehlers-Danlos Syndrome | Condition | 4062070 |
| Classical Ehlers-Danlos Syndrome | Condition | 37395762 |
| MCAS | Condition | 37309640 |
| POTS | Condition | 4159659 |
| ME/CFS | Condition | 432738 |
| COVID-19 | Condition | 37311061 |
| Post-acute COVID-19 (Long COVID) | Condition | 705076 |
