# Supplementary material for: More common than we thought: Hypermobile Ehlers-Danlos Syndrome, comorbidity burden, and long COVID risk in a national cohort
Source: PLOS Digit Health. 2026 Nov 5;5(11):e0001707. doi: 10.1371/journal.pdig.0001707 (PMC13739140; doi:10.1371/journal.pdig.0001707)
Supplement: Supplementary file 8 [file pdig.0001707.s002.docx]

**S2 Table: Logic Liaison Conditions** [[31]](https://paperpile.com/c/ycyRlK/bMT8)

| Logic Liaison Table Condition | Codeset IDs |
| --- | --- |
| Obesity | 772561500 |
| Kidney Disease | 1000049286 |
| Pregnancy | 101607593 |
| Rheumatologic Disease | 437151010 |
| Tobacco Smoker | 166730099 |
| Solid Organ Blood Stem Cell Transplant | 225621426 |
| Substance Use Disorder | 1000043391 |
| Long COVID Clinic Visit | 1000032668 |
| Suspected Covid-19 | 229223655 |
| B94.8 | 783054135 |
| Cardiomyopathies | 175678032 |
| Cerebrovascular Disease | 937546319 |
| Chronic Lung Disease | 674884280 |
| Congestive Heart Failure | 1000044269 |
| Coronary Artery Disease | 313241620 |
| Dementia | 963389709 |
| Depression | 963389709 |
| Diabetes Complicated | 263424204 |
| Diabetes Uncomplicated | 689624081 |
| Down Syndrome | 859831250 |
| Heart Failure | 1000038723 |
| Hemiplegia or Paraplegia | 174172884 |
| HIV Infection | 490831143 |
| Hypertension | 211352892 |
| COVID Diagnosis | 1000012899 |
| Long COVID Diagnosis | 1000042623 |
| Multisystem Inflammatory Syndrome | 200799555 |
| Pneumonia due to COVID | 698347441 |
| Malignant Cancer | 1000073908 |
| Metastatic Solid Tumor Cancers | 1000080916 |
| Mild Liver Disease | 17655741 |
| Moderate to Severe Liver Disease | 285109253 |
| Myocardial Infarctions | 944073846 |
| Autoimmune Disease/Immunodeficiency | 1000079426 |
| Peptic Ulcer | 1000089753 |
| Peripheral Vascular Disease | 1000020459 |
| Psychosis | 361905128 |
| Pulmonary Embolism | 503985771 |
| Sickle Cell Disease | 1000028033 |
| Substance Abuse | 606624084 |
| Thalassemia | 1000047917 |
| Tuberculosis | 772103963 |
| Nirmatrelvir | 399252964 |
| Ritonavir | 329050933 |
| Paxlovid | 123908016 |
| Remdesivir | 719693192 |
| N3C Corticosteroids for Systemic Use | 363331988 |
| Antibody Positive | 871267146, 1000005668 |
| Antibody Negative | 871267146, 1000061534 |
| PCR AG Positive | 1000019497, 1000005668 |
| PCR AG Negative | 1000019497, 1000061534 |
