# Supplementary material for: More common than we thought: Hypermobile Ehlers-Danlos Syndrome, comorbidity burden, and long COVID risk in a national cohort
Source: PLOS Digit Health. 2026 Nov 5;5(11):e0001707. doi: 10.1371/journal.pdig.0001707 (PMC13739140; doi:10.1371/journal.pdig.0001707)
Supplement: Supplementary file 9 [file pdig.0001707.s003.docx]

**S3 Table : Covariates of Cases and Controls**

| **Variable** | **hEDS** | **Control** | **Standard Deviation hEDS** | **Standard Deviation Control** |
| --- | --- | --- | --- | --- |
| **Age** | 37.23 | 37.60 | 15.60 | 21.66 |
| **Data partner: 1** | 516 | 488 |  |  |
| **Data partner: 10** | 990 | 1218 |  |  |
| **Data partner: 11** | 39 | 51 |  |  |
| **Data partner: 12** | 647 | 430 |  |  |
| **Data partner: 13** | 54 | 91 |  |  |
| **Data partner: 14** | 359 | 328 |  |  |
| **Data partner: 15** | 287 | 213 |  |  |
| **Data partner: 16** | 413 | 422 |  |  |
| **Data partner: 17** | 511 | 630 |  |  |
| **Data partner: 18** | 237 | 116 |  |  |
| **Data partner: 19** | 1274 | 687 |  |  |
| **Data partner: 2** | 201 | 203 |  |  |
| **Data partner: 20** | 209 | 162 |  |  |
| **Data partner: 21** | 262 | 185 |  |  |
| **Data partner: 22** | 587 | 346 |  |  |
| **Data partner: 23** | 22 | 116 |  |  |
| **Data partner: 24** | 441 | 626 |  |  |
| **Data partner: 25** | <20 | 71 |  |  |
| **Data partner: 26** | 124 | 117 |  |  |
| **Data partner: 27** | 316 | 687 |  |  |
| **Data partner: 28** | 889 | 1149 |  |  |
| **Data partner: 29** | 216 | 179 |  |  |
| **Data partner: 3** | 82 | 47 |  |  |
| **Data partner: 30** | 151 | 107 |  |  |
| **Data partner: 31** | 1426 | 527 |  |  |
| **Data partner: 32** | 471 | 295 |  |  |
| **Data partner: 33** | 849 | 307 |  |  |
| **Data partner: 34** | 2194 | 1537 |  |  |
| **Data partner: 35** | 143 | 195 |  |  |
| **Data partner: 36** | 242 | 342 |  |  |
| **Data partner: 37** | 187 | 238 |  |  |
| **Data partner: 38** | 218 | 346 |  |  |
| **Data partner: 39** | 644 | 1407 |  |  |
| **Data partner: 4** | 41 | 63 |  |  |
| **Data partner: 40** | 572 | 445 |  |  |
| **Data partner: 41** | 1478 | 1532 |  |  |
| **Data partner: 42** | 154 | 154 |  |  |
| **Data partner: 43** | 23 | 36 |  |  |
| **Data partner: 44** | 848 | 637 |  |  |
| **Data partner: 45** | 75 | 96 |  |  |
| **Data partner: 46** | 131 | 175 |  |  |
| **Data partner: 47** | 171 | 561 |  |  |
| **Data partner: 48** | 249 | 502 |  |  |
| **Data partner: 49** | 383 | 307 |  |  |
| **Data partner: 5** | 78 | 313 |  |  |
| **Data partner: 50** | 248 | 205 |  |  |
| **Data partner: 51** | 361 | 190 |  |  |
| **Data partner: 52** | 155 | 101 |  |  |
| **Data partner: 53** | 95 | 145 |  |  |
| **Data partner: 54** | 287 | 182 |  |  |
| **Data partner: 55** | 123 | 173 |  |  |
| **Data partner: 56** | 41 | 410 |  |  |
| **Data partner: 57** | 465 | 539 |  |  |
| **Data partner: 58** | 655 | 731 |  |  |
| **Data partner: 59** | 701 | 688 |  |  |
| **Data partner: 6** | 157 | 204 |  |  |
| **Data partner: 60** | 489 | 385 |  |  |
| **Data partner: 7** | 515 | 511 |  |  |
| **Data partner: 8** | 241 | 70 |  |  |
| **Data partner: 9** | 1209 | 1934 |  |  |
| **ED visits** | 2.93 | 2.73 | 6.85 | 8.91 |
| **Inpatient visits** | 0.99 | 0.98 | 3.30 | 3.70 |
| **Outpatient visits** | 109.46 | 104.42 | 132.66 | 163.51 |
| **Race/Ethnicity: American Indian or Alaska Native Non-Hispanic** | 143 | 163 |  |  |
| **Race/Ethnicity: Asian Non-Hispanic** | 254 | 226 |  |  |
| **Race/Ethnicity: Black or African American Non-Hispanic** | 657 | 611 |  |  |
| **Race/Ethnicity: Hispanic or Latino Any Race** | 1381 | 1181 |  |  |
| **Race/Ethnicity: Native Hawaiian or Other Pacific Islander Non-Hispanic** | 34 | 29 |  |  |
| **Race/Ethnicity: Other Non-Hispanic** | 254 | 200 |  |  |
| **Race/Ethnicity: Unknown** | 1001 | 889 |  |  |
| **Race/Ethnicity: White Non-Hispanic** | 21428 | 21853 |  |  |
| **Race: American Indian or Alaska Native** | 175 | 198 |  |  |
| **Race: Asian** | 257 | 229 |  |  |
| **Race: Black or African American** | 686 | 644 |  |  |
| **Race: Hispanic or Latino** | <20 | <20 |  |  |
| **Race: Native Hawaiian or Other Pacific Islander** | 38 | 32 |  |  |
| **Race: Other** | 349 | 301 |  |  |
| **Race: Unknown** | 1428 | 1257 |  |  |
| **Race: White** | 22218 | 22491 |  |  |
| **Sex: FEMALE** | 21180 | 21607 |  |  |
| **Sex: MALE** | 3214 | 2830 |  |  |
| **Sex: No matching concept** | 749 | 709 |  |  |
| **Sex: UNKNOWN** | <20 | <20 |  |  |
| **Total visits** | 134.01 | 126.68 | 152.35 | 186.26 |
