# Supplementary material for: More common than we thought: Hypermobile Ehlers-Danlos Syndrome, comorbidity burden, and long COVID risk in a national cohort
Source: PLOS Digit Health. 2026 Nov 5;5(11):e0001707. doi: 10.1371/journal.pdig.0001707 (PMC13739140; doi:10.1371/journal.pdig.0001707)
Supplement: Supplementary file 10 [file pdig.0001707.s004.docx]

**S4 Table: Odds ratio values and statistics**

| **condition_name** | **odds_ratio** | **lower_CI** | **upper_CI** | **p_value** | **p_value_FDR** |
| --- | --- | --- | --- | --- | --- |
| **Antibody_Pos_indicator** | 0.69 | 0.66 | 0.71 | 4.00E-71 | 3.47E-70 |
| **Antibody_Neg_indicator** | 1.14 | 1.09 | 1.19 | 7.01E-08 | 1.61E-07 |
| **PCR_AG_Pos_indicator** | 0.69 | 0.66 | 0.71 | 4.00E-71 | 3.47E-70 |
| **TOBACCOSMOKER_indicator** | 1.08 | 1.03 | 1.13 | 7.01E-04 | 0.001333912 |
| **SOLIDORGANORBLOODSTEMCELLTRANSPLANT_indicator** | 0.57 | 0.49 | 0.65 | 2.09E-16 | 7.42E-16 |
| **SUBSTANCEUSEDISORDER_indicator** | 1.16 | 1.11 | 1.21 | 3.41E-12 | 9.49E-12 |
| **MODERATESEVERELIVERDISEASE_indicator** | 0.48 | 0.41 | 0.57 | 2.68E-17 | 1.10E-16 |
| **MYOCARDIALINFARCTION_indicator** | 0.64 | 0.57 | 0.71 | 4.69E-17 | 1.74E-16 |
| **OTHERIMMUNOCOMPROMISED_indicator** | 2.05 | 1.94 | 2.15 | 3.36E-168 | 6.56E-167 |
| **DOWNSYNDROME_indicator** | 0.38 | 0.23 | 0.62 | 1.36E-04 | 2.66E-04 |
| **HEARTFAILURE_indicator** | 0.60 | 0.55 | 0.65 | 3.15E-38 | 1.89E-37 |
| **HEMIPLEGIAORPARAPLEGIA_indicator** | 1.04 | 0.90 | 1.19 | 0.62066735 | 0.880219151 |
| **CORONARYARTERYDISEASE_indicator** | 0.62 | 0.57 | 0.67 | 3.62E-34 | 2.02E-33 |
| **DEMENTIA_indicator** | 0.46 | 0.37 | 0.56 | 4.93E-14 | 1.54E-13 |
| **DEPRESSION_indicator** | 2.23 | 2.15 | 2.32 | 7.23E-24 | 4.38E-23 |
| **B94_8_indicator** | 2.41 | 1.90 | 3.05 | 4.24E-13 | 1.22E-12 |
| **CARDIOMYOPATHIES_indicator** | 0.66 | 0.59 | 0.74 | 8.18E-14 | 2.45E-13 |
| **CEREBROVASCULARDISEASE_indicator** | 1.11 | 1.03 | 1.20 | 0.005526243 | 0.009796521 |
| **PSYCHOSIS_indicator** | 1.09 | 0.96 | 1.23 | 0.196440821 | 0.306447681 |
| **PULMONARYEMBOLISM_indicator** | 1.04 | 0.92 | 1.18 | 0.500061018 | 0.735938856 |
| **SICKLECELLDISEASE_indicator** | 0.53 | 0.29 | 0.96 | 0.03683679 | 0.062462384 |
| **LL_MISC_indicator** | 0.69 | 0.20 | 2.44 | 0.566103686 | 0.817705324 |
| **LL_PNEUMONIADUETOCOVID_indicator** | 0.66 | 0.56 | 0.78 | 9.54E-07 | 2.13E-06 |
| **MALIGNANTCANCER_indicator** | 0.67 | 0.63 | 0.72 | 5.43E-34 | 2.82E-33 |
| **METASTATICSOLIDTUMORCANCERS_indicator** | 0.18 | 0.08 | 0.38 | 7.02E-06 | 1.48E-05 |
| **MILDLIVERDISEASE_indicator** | 1.04 | 0.98 | 1.11 | 0.156213894 | 0.253847578 |
| **LL_Long_COVID_clinic_visit_indicator** | 2.98 | 2.18 | 4.08 | 7.36E-12 | 1.98E-11 |
| **LL_SUSPECTEDCOVID19_indicator** | 0.78 | 0.56 | 1.11 | 0.165595327 | 0.263600725 |
| **PAX1_NIRMATRELVIR_indicator** | 1.09 | 0.92 | 1.28 | 0.312454918 | 0.468682377 |
| **PAX2_RITONAVIR_indicator** | 1.10 | 0.94 | 1.30 | 0.232402901 | 0.355439731 |
| **PAXLOVID_indicator** | 2.05 | 1.89 | 2.22 | 5.43E-70 | 4.24E-69 |
| **confirmed_covid_patient** | 0.89 | 0.86 | 0.92 | 2.52E-10 | 6.55E-10 |
| **possible_covid_patient** | 2.93 | 2.44 | 3.51 | 3.28E-31 | 1.50E-30 |
| **patient_death_indicator** | 0.43 | 0.37 | 0.49 | 3.29E-32 | 1.60E-31 |
| **CHRONICLUNGDISEASE_indicator** | 1.96 | 1.88 | 2.04 | 1.55E-233 | 4.03E-232 |
| **CONGESTIVEHEARTFAILURE_indicator** | 0.42 | 0.38 | 0.46 | 3.01E-63 | 1.96E-62 |
| **PEPTICULCER_indicator** | 1.54 | 1.39 | 1.70 | 3.28E-17 | 1.28E-16 |
| **PERIPHERALVASCULARDISEASE_indicator** | 0.75 | 0.68 | 0.82 | 3.20E-09 | 7.79E-09 |
| **REMDISIVIR_indicator** | 0.63 | 0.53 | 0.76 | 1.48E-06 | 3.21E-06 |
| **SYSTEMICCORTICOSTEROIDS_indicator** | 2.10 | 2.02 | 2.19 | 1.38E-289 | 5.38E-288 |
| **LL_COVID_diagnosis_indicator** | 1.06 | 1.02 | 1.11 | 0.001947966 | 0.00353352 |
| **LL_Long_COVID_diagnosis_indicator** | 2.88 | 2.55 | 3.26 | 2.57E-64 | 1.82E-63 |
| **DIABETESCOMPLICATED_indicator** | 0.49 | 0.46 | 0.53 | 7.63E-82 | 9.92E-81 |
| **DIABETESUNCOMPLICATED_indicator** | 0.59 | 0.55 | 0.62 | 3.44E-76 | 3.83E-75 |
| **PREGNANCY_indicator** | 0.89 | 0.85 | 0.94 | 4.79E-05 | 9.57E-05 |
| **RHEUMATOLOGICDISEASE_indicator** | 2.09 | 1.97 | 2.21 | 1.90E-134 | 2.96E-133 |
| **SUBSTANCEABUSE_indicator** | 0.88 | 0.81 | 0.95 | 0.001144735 | 0.002125936 |
| **THALASSEMIA_indicator** | 1.63 | 1.02 | 2.61 | 0.041400011 | 0.068706401 |
| **TUBERCULOSIS_indicator** | 5.20 | 3.69 | 7.33 | 3.97E-21 | 1.72E-20 |
| **MOLNUPIRAVIR_indicator** | 1.48 | 1.23 | 1.79 | 4.43E-05 | 9.09E-05 |
| **HIVINFECTION_indicator** | 0.71 | 0.51 | 0.98 | 0.036257001 | 0.062462384 |
| **HYPERTENSION_indicator** | 0.84 | 0.81 | 0.88 | 2.74E-15 | 9.30E-15 |
| **OBESITY_indicator** | 1.12 | 1.08 | 1.16 | 2.15E-09 | 5.40E-09 |
| **KIDNEYDISEASE_indicator** | 0.78 | 0.74 | 0.84 | 3.90E-14 | 1.27E-13 |
| **PCR_AG_Neg_indicator** | 1.14 | 1.09 | 1.19 | 7.01E-08 | 1.61E-07 |
