# Supplementary material for: More common than we thought: Hypermobile Ehlers-Danlos Syndrome, comorbidity burden, and long COVID risk in a national cohort
Source: PLOS Digit Health. 2026 Nov 5;5(11):e0001707. doi: 10.1371/journal.pdig.0001707 (PMC13739140; doi:10.1371/journal.pdig.0001707)
Supplement: Supplementary file 11 [file pdig.0001707.s005.docx]

**S5 Table: Prevalence Rates of Comorbidities Segmented by Long COVID Flag**

| **eds_flag** | **long_covid_flag** | **mcas_prevalence** | **pots_prevalence** | **mecfs_prevalence** | **mcas_pots_prevalence** | **mcas_mecfs_prevalence** | **pots_mecfs_prevalence** | **all_three_prevalence** |
| --- | --- | --- | --- | --- | --- | --- | --- | --- |
| **1** | 0 | 9.5 | 21.7 | 14.6 | 5.3 | 2.8 | 5.2 | 1.7 |
| **1** |  | <20 | <20 | <20 | <20 | <20 | <20 | <20 |
| **1** | 1 | 25.3 | 42.7 | 42.0 | 19.4 | 14.1 | 21.8 | 11.9 |
| **0** | 0 | <20 | <20 | 3.3 | <20 | <20 | <20 | <20 |
| **0** | 1 | <20 | <20 | 22.84 | <20 | <20 | <20 | <20 |
| **0** |  | <20 | <20 | <20 | <20 | <20 | <20 | <20 |
