# Supplementary material for: More common than we thought: Hypermobile Ehlers-Danlos Syndrome, comorbidity burden, and long COVID risk in a national cohort
Source: PLOS Digit Health. 2026 Nov 5;5(11):e0001707. doi: 10.1371/journal.pdig.0001707 (PMC13739140; doi:10.1371/journal.pdig.0001707)
Supplement: Supplementary file 12 [file pdig.0001707.s006.docx]

**S6 Table: Cumulative incidence of hEDS diagnoses by comorbidity group and index condition.**

| **Index Condition** | **Comorbidity Group** | **Day 180 Cum. Incidence (%)** | **Day 365 Cum. Incidence (%)** | **Day 730 Cum. Incidence (%)** | **Log-rank p vs hEDS+POTS** | **Log-rank p vs hEDS+MCAS** | **Log-rank p vs hEDS+ME/CFS** | **Log-rank p vs hEDS** |
| --- | --- | --- | --- | --- | --- | --- | --- | --- |
| COVID | hEDS+POTS | 75.46 | 58.48 | 29.98 | - | 0.1179 | 0.0000 | 0.0000 |
| COVID | hEDS+MCAS | 77.19 | 55.85 | 24.36 | 0.1179 | - | 0.0000 | 0.0000 |
| COVID | hEDS+ME/CFS | 88.78 | 79.15 | 61.15 | 0.0000 | 0.0000 | - | 0.0000 |
| COVID | hEDS | 94.64 | 91.32 | 85.72 | 0.0000 | 0.0000 | 0.0000 | - |
| Long COVID | hEDS+POTS | 78.73 | 61.58 | 38.22 | - | 0.1643 | 0.0005 | 0.0000 |
| Long COVID | hEDS+MCAS | 77.78 | 55.12 | 30.85 | 0.1643 | - | 0.0000 | 0.0000 |
| Long COVID | hEDS+ME/CFS | 85.34 | 74.00 | 58.09 | 0.0005 | 0.0000 | - | 0.0000 |
| Long COVID | hEDS | 92.35 | 88.65 | 84.80 | 0.0000 | 0.0000 | 0.0000 | - |
