# Supplementary material for: More common than we thought: Hypermobile Ehlers-Danlos Syndrome, comorbidity burden, and long COVID risk in a national cohort
Source: PLOS Digit Health. 2026 Nov 5;5(11):e0001707. doi: 10.1371/journal.pdig.0001707 (PMC13739140; doi:10.1371/journal.pdig.0001707)
Supplement: Supplementary file 15 [file pdig.0001707.s009.docx]

**S3 Fig: Cumulative incidence plots show patients with hEDS have higher likelihood of receiving a diagnosis after an acute post viral infection with complications when comorbidities are present.**
