# Supplementary material for: More common than we thought: Hypermobile Ehlers-Danlos Syndrome, comorbidity burden, and long COVID risk in a national cohort
Source: PLOS Digit Health. 2026 Nov 5;5(11):e0001707. doi: 10.1371/journal.pdig.0001707 (PMC13739140; doi:10.1371/journal.pdig.0001707)
Supplement: Supplementary file 16 [file pdig.0001707.s010.docx]

**S4 Fig. Additional Top-Charted Conditions.**

| **4a** | **4b** |
| --- | --- |
| **4c** | **4d** |
| **4e** | **4f** |
