# Supplementary material for: More common than we thought: Hypermobile Ehlers-Danlos Syndrome, comorbidity burden, and long COVID risk in a national cohort
Source: PLOS Digit Health. 2026 Nov 5;5(11):e0001707. doi: 10.1371/journal.pdig.0001707 (PMC13739140; doi:10.1371/journal.pdig.0001707)
Supplement: Supplementary file 17 [file pdig.0001707.s017.docx]

**We thank the reviewers for their very constructive feedback, which we believe has strengthened the manuscript. Our detailed responses to the reviewers’ comments and suggestions are highlighted in bold, with exact text changes highlighted in blue.**

1. The title must not imply that viral infection reveals or causes the disorder. Both the "unmask the disorder" phrasing and a bare "after COVID-19" construction carry that implication for a general readership. An honest associational framing centered on diagnosis timing (e.g., hEDS diagnosis being 'associated with' prior COVID-19 documentation) is what the evidence supports, and is what the reviewer and I are asking for.

- **We apologize for the consistent implication remaining in the title after edits. To address this we changed the title from “Hypermobile Ehlers-Danlos Syndrome Is Diagnosed More Often After COVID-19: Prevalence, Comorbidities, and Diagnostic Timing” to “More Common Than We Thought: Hypermobile Ehlers-Danlos Syndrome, Comorbidity Burden, and Long COVID Risk in a National Cohort” We feel this more accurately supports the main conclusions from the paper; that hEDS is more common than previously recorded, that specific comorbid conditions are enriched in hEDS patients, and that patients with hEDS and comorbid conditions have higher rates of Long Covid.**

2. Abstract, discussion, and conclusions must be reframed throughout as a descriptive study of comorbidity patterns, diagnostic timing, and healthcare utilisation in hEDS. Where the "unmasking" mechanism is raised, it should appear explicitly as one of at least two equally supported hypotheses, not as the favored reading with alternatives appended as caveats. The current text hedges in places but still leads with biology; the balance needs to shift so that the ascertainment explanation is given genuine parity.

**We apologize for not fully addressing this in the last round of revisions and hope the revisions below suffice to reframe the manuscript throughout. Additionally, in a few places, we found it inappropriate to point towards mechanistic explanations, rather than other underlying associations present in the data. We adjusted where necessary.**

- **In the abstract we replaced “Temporal diagnostic analyses revealed that many patients received an hEDS diagnosis only after a COVID-19 infection, consistent with viral illness unmasking previously unrecognized connective tissue dysfunction, increased post-viral healthcare engagement bringing a longstanding diagnosis to light, or both.” with “A subgroup of patients received their hEDS diagnosis only after a COVID-19 or Long COVID diagnosis, and this subgroup was highly enriched for multimorbidity, particularly POTS and MCAS.”. We chose to keep the evidence for enrichment of an hEDS diagnosis post covid from those with multimorbidity burden, as that is what the data shows, and keep speculation out of the abstract.**
- **Additionally, in the Author Summary, we replaced “In many cases, people were diagnosed with hEDS for the first time only after they had COVID-19, suggesting that viral illness may worsen or reveal underlying symptoms, or that increased medical care following infection finally brings a longstanding but unrecognized condition to light.” with “A subgroup of patients were diagnosed with hEDS for the first time only after having COVID-19 or Long COVID, with high rates of POTS and MCAS.” Again, this is highlighting the findings without raising any biological mechanisms to the reader.**
- **In the Introduction, we replaced "Anecdotal and clinical observations suggest that SARS-CoV-2 infection may exacerbate or unmask underlying hEDS symptoms, triggering a new diagnosis. We aimed to determine whether viral infection acts as a diagnostic inflection point for hEDS, and to assess the extent to which biological unmasking and increased healthcare utilization each contribute to the observed temporal patterns." with "Anecdotal and clinical observations suggest that SARS-CoV-2 infection may be associated with new or worsening hEDS symptoms in some patients. We aimed to characterize the prevalence, comorbidity burden, and Long COVID risk associated with hEDS, and to describe the timing of hEDS diagnosis relative to COVID-19 and Long COVID in this population."**
- **Later in the same section, we replaced "...characterize key comorbidities, and assess whether viral infection may act as a diagnostic inflection point for previously unrecognized hEDS." with "...characterize key comorbidities, and describe the timing of hEDS diagnosis relative to COVID-19 infection in this population." These changes were made to align the stated aims of the study with the framing used throughout the rest of the revised manuscript.**
- **In the results section Titled Patients with hEDS are more likely to receive a diagnosis after COVID-19 and Long COVID when comorbid conditions are present, we replaced “These findings support the hypothesis that a viral infection might act as a physiological stressor, unmasking latent connective tissue dysfunction in genetically susceptible individuals, though increased healthcare utilization following infection may contribute equally to this pattern.” with “Overall, these results indicate that the elevated likelihood of an hEDS diagnosis following COVID-19 or Long COVID is concentrated among patients with comorbid POTS or MCAS, rather than occurring uniformly across the hEDS population.” The main conclusion of this section is the title, so we focused on the main data point from this section rather than describing why this may be occurring.**
- **Within the same results section, we removed “Together, these findings suggest that overlapping symptom syndromes, especially POTS and MCAS, may reveal latent connective tissue disorders in the post-infectious setting, whether through biological unmasking or increased diagnostic engagement driven by post-viral care-seeking.” because we feel the data more accurately portrays the previous sentence “that autonomic dysfunction and immune dysregulation are the primary factors driving accelerated hEDS diagnosis following COVID-19,” and we don’t need to point towards a reason here.**
- **Within the Discussion paragraph 2, we replaced “This could reflect symptom exacerbation, underlying pathophysiological overlap, or increased healthcare engagement and diagnostic attention following infection.” with “Despite similar rates of acute infection, patients with hEDS were nearly three times more likely to receive a Long COVID diagnosis, indicating that hEDS elevates risk specifically for the post-acute phase of illness rather than for infection itself.” to focus more on the Long Covid risk in some hEDS individuals.**
- **Additionally, in the same paragraph, we replaced “Overall, these findings suggest that patients with hEDS are particularly vulnerable to developing Long COVID after a COVID-19 infection, which we speculate could be due to symptom exacerbation or underlying pathophysiological overlap, though alternative explanations cannot be excluded.” with “Overall, these findings establish that patients with hEDS carry a substantially elevated burden of Long COVID relative to matched controls, a pattern that holds whether cases are identified through the dedicated Long COVID code or the broader B94.8 sequelae code.” We feel the data more accurately supports this conclusion.**
- **In the Discussion paragraph 4, we replaced “The increase in raw counts among post-COVID patients is therefore driven by encounter volume rather than a shift in diagnostic yield per visit. Whether that increased encounter volume reflects passive healthcare-seeking following infection or symptom-driven care-seeking in patients whose hEDS was biologically triggered or worsened by COVID-19 remains an open question, and one these data cannot resolve.” with “The increase in raw counts among post-COVID patients is therefore driven by encounter volume rather than a shift in diagnostic yield per visit, which specifically argues against a model in which clinicians became more likely to diagnose hEDS at a given visit following COVID-19. This does not, however, distinguish between passive healthcare-seeking unrelated to hEDS and symptom-driven care-seeking in patients whose hEDS was biologically triggered or worsened by COVID-19, since both pathways would produce the same pattern of increased encounter volume without a corresponding rise in per-visit diagnostic yield. Distinguishing definitively between these explanations would require additional studies in future work.” We believe this more precisely characterizes what the encounter-adjusted analysis can and cannot establish: it rules out increased diagnostic vigilance per visit, but it does not, on its own, favor an ascertainment explanation over a genuine biological pathway involving increased symptom-driven care-seeking following infection.**
- **In Discussion paragraph 5, we replaced “This pattern is consistent with both biological unmasking and increased clinical investigation following infection, prompting eventual recognition of hEDS. For example, post-viral worsening of autonomic symptoms or immune dysregulation may lead clinicians to consider diagnoses such as POTS or MCAS, which can then reveal the underlying connective tissue disorder. However, alternative explanations, including increased healthcare contact and diagnostic attention, remain plausible.” with “Taken together with the comorbidity enrichment noted above, this suggests that MCAS and POTS mark a subgroup of patients with hEDS who are both more likely to be diagnosed following infection and more likely to develop Long COVID, consistent with these conditions conferring added vulnerability to prolonged post-viral illness.” We think this more accurately portrays the results of the data provided, while not drawing conclusions to the ‘why’.**
- **We also revised paragraph 6. We added, "It is equally consistent with an ascertainment pathway, in which patients already managing MCAS- or POTS-related symptoms have more frequent contact with the healthcare system following infection, increasing the likelihood that a pre-existing but uncoded hEDS presentation is captured in the record." We retained the biological hypothesis in full and added the ascertainment explanation as an equally developed alternative, consistent with genuine parity between the two interpretations rather than favoring one as the primary reading.**
- **Finally, in the conclusions, we rewrote the conclusion entirely to point towards the main evidence given in the paper. We chose to leave out the speculative portion entirely. Shown below are the specific claims from the conclusion section:**
  - **“Despite similar rates of COVID-19 infection, patients with hEDS were nearly three times more likely to develop Long COVID, and this risk was concentrated among those with comorbid MCAS, POTS, and/or ME/CFS. Patients with hEDS also carried significantly elevated rates of rheumatologic, autoimmune, and cerebrovascular disease, underscoring the systemic complexity of hEDS and its potential interactions with immune and autonomic pathways. Cumulative incidence analyses revealed that a subgroup of hEDS diagnoses occurred after COVID-19 and Long COVID, with the highest rates among patients with POTS and MCAS, conditions rooted in autonomic and immune dysregulation. In these cases, a viral infection may mark the inflection point for the clinical recognition of a connective tissue disorder.”**

3. Cohort comparability (Figure 3). Please address directly what the protective-direction odds ratios - death (0.43), pneumonia (0.66), positive PCR (0.69) - imply about the matched comparison. As one reviewer noted, differences of this magnitude suggest residual differences in baseline health, healthcare access, testing behavior, or surveillance intensity between cohorts, and this bears on how much interpretive weight the matched associations can carry. This should be discussed as a limitation on the comparison itself, not only as a treatment-pattern observation.

- **We replaced all of paragraph 3 in the Discussion “We also found that patients with hEDS were more likely to receive Paxlovid but significantly less likely to receive Remdesivir, which is administered intravenously and used primarily in inpatient settings. Consistent with this, patients with hEDS had lower odds of pneumonia and death following COVID-19. Together, these patterns suggest that patients with hEDS may be more likely to receive early outpatient treatment, potentially preventing disease progression to hospitalization, though residual differences in baseline health status and healthcare access between the matched cohorts could also contribute to these findings. This pattern may reflect a higher likelihood of moderate complications requiring attention, differences in provider prescribing practices, or the relative accessibility of oral versus intravenous treatments. Additionally, Paxlovid became available in late 2021, as awareness of Long COVID risk increased, so clinicians might preferentially prescribe Paxlovid to patients with known chronic conditions such as hEDS. To fully understand these results, further investigation into hospitalization rates or ICU admissions would be necessary.” with “We also found that patients with hEDS were more likely to receive Paxlovid but significantly less likely to receive Remdesivir, and had lower odds of pneumonia and death following COVID-19. This pattern is consistent with earlier outpatient antiviral treatment preventing progression to more severe illness, differences in provider prescribing practices, or the relative accessibility of oral versus intravenous therapies. Paxlovid became available in late 2021, as awareness of Long COVID risk increased, so clinicians may have preferentially prescribed it to patients with known chronic conditions such as hEDS. Further investigation into hospitalization and ICU admission rates would help clarify these patterns.” Summarizing the findings better, while leaving pertinent information about limitations for the limitations section.**
- **In the limitations section, paragraph 4, we replaced “these explanations; in their absence, these findings are best understood as descriptive and hypothesis-generating.” with “the biological-unmasking and ascertainment explanations for our timing findings. Separately, several protective-direction associations in the matched comparison itself including lower odds of death (OR = 0.43), pneumonia (OR = 0.66), and positive PCR testing (OR = 0.69) among patients with hEDS, are large enough to suggest residual differences in baseline health, testing behavior, or surveillance intensity between the matched cohorts, despite matching on demographics, healthcare utilization, and site. This bears on the interpretive weight of the matched-cohort associations reported throughout the manuscript, including those in Figures 3 and 7. In the absence of chart validation, an independent comparator, and full assurance of cohort comparability, we interpret all matched-cohort findings, including the diagnostic-timing results, as descriptive and hypothesis-generating rather than causal.” We feel this addresses the reviewer’s comment regarding potential residual differences in the propensity-matched cohort.**

4. The summary statement that testing-related variables, pneumonia, and mortality showed no difference between groups is inconsistent with the revised Figure 3, where several of these do differ. Please correct this and any related passages so the narrative matches the current results.

- **We believe this was addressed in a previous round of revisions and remains corrected in the current manuscript. The Results section accurately reports the significant lower odds observed: "In contrast, patients with hEDS had significantly lower odds of Remdesivir use (OR = 0.63, FDR = 3.2e-6), positive PCR test (OR = 0.69, FDR < 1e-10), positive antibody test (OR = 0.69, FDR < 1e-10), pneumonia following COVID-19 (OR = 0.66, FDR = 2.1e-6), and death (OR = 0.43, FDR < 1e-10)." We no longer find any statement in the manuscript claiming these variables showed no difference between groups. As described in our response to point 3, we have separately expanded our interpretation of these protective-direction associations in the Discussion and Limitations, addressing what they imply about the comparability of the matched cohorts. If the editor is referring to a specific passage that still requires correction, we would welcome it being identified so we can address it directly.**
