# Supplementary material for: More common than we thought: Hypermobile Ehlers-Danlos Syndrome, comorbidity burden, and long COVID risk in a national cohort
Source: PLOS Digit Health. 2026 Nov 5;5(11):e0001707. doi: 10.1371/journal.pdig.0001707 (PMC13739140; doi:10.1371/journal.pdig.0001707)
Supplement: Supplementary file 18 [file pdig.0001707.s018.docx]

**Response to reviewers V5**

**We thank the editor and reviewers for their careful reading and for identifying these inconsistencies. We sincerely apologize for these errors. Several were copy-paste/transcription mistakes, and others stemmed from the substantial re-analysis performed between the first and second submissions, when the underlying database and cohort changed and not every downstream number, table, and figure was updated to match. We have corrected each item below, outlined in bold.**

1) The cohort size doesn't reconcile. Methods gives 7,786 + 24,031 - 5,709 overlap = 26,108, then removes 216 dual-coded patients and states the final cohort is 25,192. That subtraction gives 25,892. Meanwhile every column in Table 2 (sex, ethnicity, race) sums to 25,152, and the Figure 6 and Supplementary Figure 1 legends also use 25,152, while Table 1, Table 2's header and the Figure 2 legend use 25,192 or 26,108. The Figure 2 legend actually calls 26,108 the *final* cohort, directly contradicting the text. Three different denominators are circulating. This feeds the prevalence estimate, so it has to be resolved and made consistent throughout.
   a) **The correct final cohort size is 25,152. We have corrected Figure 2 and Figure 3 accordingly, and have updated every instance of this number throughout the manuscript including the Methods text, Table 1, Table 2's header, and the Figure 2 legend, so that 25,152 is used consistently as the final cohort denominator. This is now internally consistent with Table 2's demographic totals and the denominators already used in Figure 6 and Supplementary Figure 1.**
2) Table 2's age column doesn't add up. It sums to 25,089. The 76-100 row is listed as 318 (1.51%), but 318/25,152 = 1.26%; 1.51% implies ~380, which would reconcile the column. Also 0-18 and 18-25 overlap at 18, which is not acceptable.
   a) **Confirmed and corrected. The 76–100 age row was a data entry error: the correct count is 381, not 318, which reconciles the age column to the corrected total cohort of 25,152. We have also corrected the overlapping age bins so that 0–18 and 18–25 no longer share age 18.**
3) Table 4 contains a practically impossible confidence interval. POTS: HR 3.28, 95% CI 2.65-3.28 - the upper bound equals the point estimate. Repeated verbatim in the Results text. MCAS (1.82, CI 1.60-2.33) is also implausibly non-central on the log scale. Please check that this is correct.
   a) **Confirmed. This was a copy-paste error when transcribing values from our Cox proportional hazards model output. The correct values are: POTS, HR = 2.95 (95% CI: 2.65–3.28); MCAS, HR = 2.06 (95% CI: 1.82–2.33). We have corrected both Table 4 and the corresponding sentence in the Results, and have re-verified all reported hazard ratios and confidence intervals against our saved model output to confirm these are now accurate.**
4) Thalassemia is reported both ways. Results: "not statistically significant… q = 0.069." Discussion: listed among conditions with "significantly higher odds." The latter is wrong.
   a) **Confirmed and corrected. Thalassemia did not reach statistical significance after FDR correction (OR = 1.63, 95% CI: 1.02–2.61, q = 0.069), as correctly stated in the Results. We have removed thalassemia from the list of conditions described as showing "significantly higher odds" in the Discussion and revised the surrounding text to accurately reflect this as a non-significant, exploratory finding.**
5) Fix citations. Citation [30] is used for the N3C Enclave description but reference 30 is Logarbo et al. on hEDS and Long COVID - the N3C data-descriptor citation appears to be absent. [31] is cited twice in one sentence. Reference 31 itself lists the author as "agz5de," a GitHub handle.
   a) **Corrected. We have added the appropriate N3C data-descriptor citation (Haendel et al., 2021, Journal of the American Medical Informatics Association) for the description of the N3C Enclave, replacing the erroneous reference to Logarbo et al. We have removed the duplicate citation within the same sentence, and corrected the author listing for the Logic Liaison reference, replacing the GitHub username "agz5de" with the actual named contributors. All subsequent reference numbers have been updated accordingly throughout the text and reference list.**
