# Supplementary material for: More common than we thought: Hypermobile Ehlers-Danlos Syndrome, comorbidity burden, and long COVID risk in a national cohort
Source: PLOS Digit Health. 2026 Nov 5;5(11):e0001707. doi: 10.1371/journal.pdig.0001707 (PMC13739140; doi:10.1371/journal.pdig.0001707)
Supplement: Peer review history

# Peer review: Decision Letter 0

**DOI:** 10.1371/journal.pdig.0001707.r001

**Authors:** Laura Sbaffi, Umbereen Sultana Nehal

28 Oct 2025

PDIG-D-25-00524

Understanding Comorbidities in Hypermobile Ehlers-Danlos Syndrome: Could a Viral Infection Lead to a Diagnosis?

PLOS Digital Health

Dear Dr. Pearson,

Thank you for submitting your manuscript to PLOS Digital Health. After careful consideration, we feel that it has merit but does not fully meet PLOS Digital Health's publication criteria as it currently stands. Therefore, we invite you to submit a revised version of the manuscript that addresses the points raised during the review process.

Please submit your revised manuscript within 60 days Dec 27 2025 11:59PM. If you will need more time than this to complete your revisions, please reply to this message or contact the journal office at digitalhealth@plos.org. When you're ready to submit your revision, log on to https://www.editorialmanager.com/pdig/ and select the 'Submissions Needing Revision' folder to locate your manuscript file.

Please include the following items when submitting your revised manuscript:

* A rebuttal letter that responds to each point raised by the editor and reviewer(s). You should upload this letter as a separate file labeled '?>Response to ReviewersRevised Manuscript with Track ChangesManuscript**Journal Requirements:** **Additional Editor Comments (if provided):**

Please go through the provided reviews in detail. Our reviewers carefully considered your submission and found it to be a very interesting evaluation of an under-studied condition but with some deeply concerning methodological flaws. All three reviewers flagged that findings could be wrongly labeled data, bias, confounding, changed patient behavior during COVID affecting contact with the health system, or it being simple association. As a result the conclusion is is being stated far too strongly as if the analysis proves causation. For this reason, it would need major revision prior to being considered for publication.

Please see below for the detailed reviews. The most critical elements to address are:

1. Change "Could a Viral Infection Lead to a Diagnosis?" to "Is Viral Infection Associated with Increased hEDS Diagnosis?"

This is more of a hypothesis generating, not causation-proving study. It is not appropriate to conclude causation. At most can conclude association that needs further study to validate as well as to elucidate if causation or artifact. The phrase COVID-19 “acts as a physiological stressor that unmasks underlying connective tissue dysfunction.” must be presented as one of several hypothesis. This type of data and analysis -- lacking clinical note validation or lab values -- are not appropriate to make conclusions about physiological processes.

2. Lack of a control. This could be achieved by

a-Showing temporal trends in the years before. Even so that would not account for healthcare utilization pattern changes in COVID. (Reviewer 2 suggestion)

b-Comparison to a comparable condition (Reviewer 3 suggestion)

The analysis needs to extend to both of these to better address confounding or bias

4. Provide a summary table (ideally in the Supplementary Materials) that presents the balance of major covariates between the hEDS and matched control groups.

a. Also include a new variable of site of visit or coding (specialist, ED, primary care, etc) as coding practices will differ by location and clinician type

5. Clearly address the issue of false positives and false negatives via wrong coding. The study design of Demmler et all (2019, Wales) used validated clinical criteria, not just ICD codes. This needs to be address more clearly in the discussion with clarity that this is methodologically very different from Demmler et al. The discussion should call for clinical validation as future research.

a. One way to address this and verify: a chart review sub-study of 100-200 randomly selected hEDS cases to ensure they meet diagnostic criteria

b. address the issue of B94.8 ("sequelae of infectious disease") was used as a proxy for Long COVID before U09.9 existed -- this can be addressed by extending analysis to pre-COVID years to show what the baseline was

c. address " 154 hEDS patients with "vertebral tuberculosis" versus <20 controls - an OR of 7.14. This is epidemiologically impossible and reveals a fundamental coding problem:

Vertebral TB is exceedingly rare in the US (incidence ~0.1-0.4 per 100,000)" -- how reliable are these data and what is evidence of mislabeling or coding error?

6. Clarification of Sample Counts. In Figure 5, the manuscript reports 9,279

diagnosed hEDS patients and 27,937 suspected EDS patients. However, these numbers

would sum to 37,216, while the text refers to a total of approximately 30,000

patients. The authors should clarify how these counts were derived—specifically,

whether there is overlap between the diagnosed and suspected groups, or if different

inclusion criteria or data filters were applied.

7. Missing References. On page 20, references [32] and [33], which appear to

correspond to software used in the analysis, are missing. Please ensure that all

cited tools and packages are properly referenced.

8. hEDS prevalence:It would be more informative and statistically appropriate to include confidence intervals, both for the current

estimate and for previously published ones. This would allow readers to assess whether the observed difference is statistically or substantively significant.

**Reviewers' Comments:**

**Comments to the Author**

1. Does this manuscript meet PLOS Digital Health’s publication criteria?

Reviewer #1: Yes

Reviewer #2: No

Reviewer #3: Partly

**********

2. Has the statistical analysis been performed appropriately and rigorously?

Reviewer #1: I don't know

Reviewer #2: No

Reviewer #3: Yes

**********

3. Have the authors made all data underlying the findings in their manuscript fully available (please refer to the Data Availability Statement at the start of the manuscript PDF file)?

The PLOS Data policy

Reviewer #1: Yes

Reviewer #2: No

Reviewer #3: Yes

**********

4. Is the manuscript presented in an intelligible fashion and written in standard English?

Reviewer #1: Yes

Reviewer #2: Yes

Reviewer #3: Yes

**********

Reviewer #1: The article presents novel epidemiologic findings from the N3C COVID-19 Enclave database, suggesting an unexpectedly high prevalence of hEDS and a potential association with COVID. However, certain methodological limitations may compromise the reliability of some conclusions, which require clarification and reconsideration.

1. Prevalence Estimation Biases: The reported higher prevalence of hEDS (~1 in 800) in N3C could be due to selection bias, the N3C cohort overrepresents individuals with greater healthcare utilization such as hEDS.

2. Mislabeling of related conditions, especially dysautonomia, POTS, and MCAS as hEDS is a very common practice. Therefore, considering nonspecific/General EDS as hEDS is highly plausible, specifically during the COVID-19 pandemic and overwhelmed healthcare systems. In addition, information bias caused by inconsistent documentation and variable use of diagnostic codes across institutions further degrade the diagnostic fidelity of hEDS within registry data. These biases should be considered at limitation section.

3. The inclusion of phenotypes such as infertility, autism spectrum disorder, structural gastrointestinal anomalies, and movement disorders as hEDS-related phenotypes lacks supportive evidence. While such associations may be worthy of hypothesis generation, their presentation as established findings is premature and potentially misleading.

4. Several explicit claims in the discussion fall outside the scope of the data presented. For example: “The elevated odds of tuberculosis, for instance, may reflect increased susceptibility due to immune dysregulation or preexisting lung pathology in hEDS patients.” “The increased use of systemic corticosteroids is also notable, given their known adverse effects on connective tissue integrity.” or “Finally, the elevated odds of hemiplegia or paraplegia in the hEDS population may reflect neurological complications stemming from central nervous system fragility or spinal instability, which are increasingly recognized as potential hEDS manifestations.”

Reviewer #2: Detailed concerns are provided in attached review, and addendum containing concerns with statistical methods.

Reviewer #3: Please see the attached pdf file for details. Overall, this is a clearly written and well-organized manuscript that addresses an important topic. I provide several major and minor comments in the attached pdf file.

**********

what does this mean?). If published, this will include your full peer review and any attached files.

**Do you want your identity to be public for this peer review?**  If you choose “no”, your identity will remain anonymous but your review may still be made public.

For information about this choice, including consent withdrawal, please see our Privacy Policy

Reviewer #1: No

Reviewer #2: No

Reviewer #3: No

**********

**Figure resubmission:**

**Reproducibility:**

To enhance the reproducibility of your results, we recommend that authors of applicable studies deposit laboratory protocols in protocols.io, where a protocol can be assigned its own identifier (DOI) such that it can be cited independently in the future. Additionally, PLOS ONE offers an option to publish peer-reviewed clinical study protocols. Read more information on sharing protocols at https://plos.org/protocols?utm_medium=editorial-email&utm_source=authorletters&utm_campaign=protocols

Attachment Submitted filename: Critical Flaws PDIG-D-25-00524.pdf

Attachment Submitted filename: PDIG-D-25-00524-Stat Methods Concerns.pdf

Attachment Submitted filename: referee-report.pdf

# Author comment: Author response to Decision Letter 1

**DOI:** 10.1371/journal.pdig.0001707.r002

13 Feb 2026

Attachment Submitted filename: Response to Reviewer Comments.pdf

# Peer review: Decision Letter 1

**DOI:** 10.1371/journal.pdig.0001707.r003

**Authors:** Laura Sbaffi

10 Jun 2026

PDIG-D-25-00524R1

Understanding Comorbidities in Hypermobile Ehlers-Danlos Syndrome: Could a Viral Infection Unmask the Disorder?

PLOS Digital Health

Dear Dr. Pearson,

Thank you for submitting your manuscript to PLOS Digital Health. After careful consideration, we feel that it has merit but does not fully meet PLOS Digital Health's publication criteria as it currently stands. Therefore, we invite you to submit a revised version of the manuscript that addresses the points raised during the review process.

Please submit your revised manuscript by Aug 09 2026 11:59PM. If you will need more time than this to complete your revisions, please reply to this message or contact the journal office at digitalhealth@plos.org. When you're ready to submit your revision, log on to https://www.editorialmanager.com/pdig/ and select the 'Submissions Needing Revision' folder to locate your manuscript file.

Please include the following items when submitting your revised manuscript:

* A letter that responds to each point raised by the editor and reviewer(s). You should upload this letter as a separate file labeled '?>Response to ReviewersRevised Manuscript with Track ChangesManuscript**Journal Requirements:** **Additional Editor Comments (if provided):**

Thank you for submitting your revised manuscript "Understanding Comorbidities in Hypermobile Ehlers-Danlos Syndrome: Could a Viral Infection Unmask the Disorder?" (PDIG-D-25-00524R1), to PLOS Digital Health. The manuscript has been re-evaluated by the original reviewers, and I have read both their reports and your point-by-point responses carefully alongside the revised manuscript.

I want to begin by acknowledging the seriousness with which you approached this revision. This was not a cosmetic exercise. The addition of formal temporal testing through Cox proportional hazards modelling and interrupted time-series analysis, the move to conditional logistic regression for the matched comparisons, the application of false discovery rate correction across the multiple-comparison analyses, and the re-matching of controls on healthcare utilisation all represent substantive methodological strengthening. I also want to commend the decision to remove the vertebral tuberculosis finding rather than attempt to explain it away; identifying it as a likely coding artefact and reporting that transparently was the correct and honest scientific judgement. The manuscript is meaningfully tighter and more candid than the original submission.

The two reviewers have, however, reached divergent conclusions. One recommends rejection on the grounds that the central interpretation is not adequately supported by the data, while the other considers the remaining issues addressable through minor revision. Having weighed both perspectives against the revised manuscript, my decision is that the paper requires major revision before it can be considered further. I do not regard the divergence as irreconcilable: the reviewer recommending rejection has identified a genuine and currently unresolved problem, but I believe it is one that can be addressed through reframing and clearer acknowledgement of the data's limits rather than necessarily requiring new analyses that the data access situation may not permit.

The core concern, which both reviewers circle in different ways, is this. Without a comparator condition such as fibromyalgia or chronic pain, and without chart-level validation of the hEDS phenotype, the data cannot distinguish between two fundamentally different explanations: that viral infection unmasks genuine latent hEDS, or that COVID-era increases in healthcare engagement surfaced previously uncoded symptom clusters that were subsequently labelled as hEDS. These two interpretations are, on the present evidence, close to observationally equivalent. Your own encounter-adjusted analysis in Figure 4 is central here, and I would note that its interpretation cuts in a direction that complicates the central thesis. The stability of diagnosis rates per encounter, with broadly similar rates across the COVID-before, COVID-after, and never-COVID groups, is at least as consistent with a surveillance and ascertainment account as it is with biological unmasking. The manuscript currently reads this stability as arguing against ascertainment bias, but a reader could equally conclude that it provides little positive support for the unmasking hypothesis. This needs to be confronted directly rather than resolved in favour of the more mechanistic reading.

Relatedly, I must be explicit about the title, as this is a condition of any further consideration rather than a stylistic suggestion. The reviewers asked that the title be revised to make the interpretive ambiguity explicit, for example by framing the finding as an association with increased diagnosis rather than as unmasking. The current title, "Could a Viral Infection Unmask the Disorder?", retains the biological framing through its central verb even while posing it as a question. Given that the data are equally consistent with the surveillance-artefact interpretation, the title tips the reader toward the more mechanistic and less well-supported explanation before the evidence has been presented. I am asking that the title be revised to reflect what the data can actually support, namely an observed association between viral infection and increased hEDS diagnosis, without privileging a causal or mechanistic reading. This should be carried through consistently into the abstract, the author summary, the discussion, and the conclusions, where similar framing currently appears.

I recognise fully that the N3C data access constraints are real and material. A ten-day re-access window in December 2025 is a genuine limitation, and I am not asking you to conduct analyses that the enclave's availability makes impossible. Where the comparator-condition analysis or chart-level validation cannot be performed, the appropriate response is to state plainly in the discussion and limitations that the data cannot adjudicate between the unmasking and ascertainment interpretations, and to ensure the framing throughout the manuscript does not claim more than the evidence allows. The science here is valuable as a descriptive, hypothesis-generating characterisation of hEDS comorbidity patterns, diagnostic timing, and post-viral outcomes in a large EHR cohort. It does not need to assert a mechanism to make a genuine contribution, and it is on firmer ground when it does not.

In your revision and response, I would ask you specifically to:

1. Revise the title so that it describes an association with increased hEDS diagnosis rather than implying biological unmasking, and align the abstract, author summary, discussion, and conclusions with this framing.

2. Give the surveillance and diagnostic-ascertainment interpretation genuinely equal weight throughout, and reconsider the interpretation of Figure 4 so that the encounter-adjusted findings are not presented as evidence for unmasking when they are at least equally consistent with an ascertainment account.

3. State explicitly, in the discussion and limitations, that in the absence of a comparator condition and chart-level validation the data cannot distinguish between the competing explanations, and clearly position the work as descriptive and hypothesis-generating.

4. Address the consistency point raised regarding Figure 3, where the text summarising testing-related and outcome variables should be reconciled with the differences now visible in the revised figure.

Please accompany your revised manuscript with a point-by-point response addressing these points and the reviewers' full comments, which are appended below.

**Reviewers' Comments:**

**Comments to the Author**

Reviewer #2: (No Response)

Reviewer #3: (No Response)

**********

publication criteria?

Reviewer #2: Yes

Reviewer #3: No

**********

3. Has the statistical analysis been performed appropriately and rigorously?

Reviewer #2: Yes

Reviewer #3: No

**********

4. Have the authors made all data underlying the findings in their manuscript fully available (please refer to the Data Availability Statement at the start of the manuscript PDF file)?

The PLOS Data policy

Reviewer #2: Yes

Reviewer #3: Yes

**********

5. Is the manuscript presented in an intelligible fashion and written in standard English?

Reviewer #2: Yes

Reviewer #3: Yes

**********

Reviewer #2: The authors engaged substantively - this is not merely a cosmetic revision; for that, I commend them. The most important upgrades were the addition of formal temporal testing (Cox regression, interrupted time-series), switching to conditional logistic regression for matched analyses, applying FDR correction, and re-matching on healthcare utilization. Removing the vertebral TB finding was the correct and honest call.

That said, three concerns remain genuinely unresolved and they're interconnected:

The core unresolved problem is that without a fibromyalgia/chronic pain comparator, and without chart-level validation, you cannot distinguish "COVID unmasks real hEDS" from "COVID-era healthcare engagement unmasks previously uncoded symptom clusters that get labeled hEDS." The revised title - "Could a Viral Infection Unmask the Disorder?" - still implies the biological interpretation while the data are equally consistent with the surveillance artifact interpretation. The reviewers wanted the title to make that ambiguity explicit ("associated with increased diagnosis"), and that is not appropriate and not supported by their data.

The N3C data access situation is a real constraint - a 10-day window in December 2025 is genuinely limited - but it means some of the most important analytical work simply couldn't be done.

The paper is more honest and methodologically tighter than the original, but the fundamental conflicting interpretations remain unresolved by the data, and the title still attempts to tip the framing toward the more exciting (and less supported) explanation.

The title must be honestly revised before acceptance: their implication is not supported by their data.

Reviewer #3: Please see the attached pdf.

**********

what does this mean?). If published, this will include your full peer review and any attached files.

**Do you want your identity to be public for this peer review?**  If you choose “no”, your identity will remain anonymous but your review may still be made public.

For information about this choice, including consent withdrawal, please see our Privacy Policy

Reviewer #2: No

Reviewer #3: No

**********

**Figure resubmission:**

**Reproducibility:**

To enhance the reproducibility of your results, we recommend that authors of applicable studies deposit laboratory protocols in protocols.io, where a protocol can be assigned its own identifier (DOI) such that it can be cited independently in the future. Additionally, PLOS ONE offers an option to publish peer-reviewed clinical study protocols. Read more information on sharing protocols at https://plos.org/protocols?utm_medium=editorial-email&utm_source=authorletters&utm_campaign=protocols

Attachment Submitted filename: report-redacted.pdf

# Author comment: Author response to Decision Letter 2

**DOI:** 10.1371/journal.pdig.0001707.r004

6 Jul 2026

Attachment Submitted filename: Response to reviewer Comments V3.pdf

# Editor report: Decision Letter 2

**DOI:** 10.1371/journal.pdig.0001707.r005

**Authors:** Laura Sbaffi

8 Jul 2026

Response to ReviewersRevised Manuscript with Track ChangesManuscript**Journal Requirements:** **Additional Editor Comments (if provided):**

Thank you for your revised submission and for the substantial work behind it. I have now considered the revised manuscript alongside the returning reviewer's report and have read the paper myself in detail. I am pleased to say this is a genuine, substantive revision rather than a cosmetic one, and several of the changes materially strengthen the study. In particular, the move to conditional logistic regression for the matched analyses, the addition of Benjamini-Hochberg FDR correction, the re-matching on healthcare utilisation, and the new formal temporal analyses (Cox proportional hazards and the interrupted time-series) all address prior methodological concerns directly. Removing the vertebral tuberculosis signal, with a transparent explanation, was the correct call.

Despite these improvements, one concern remains and it is central enough that I cannot accept the manuscript in its current framing. The data cannot distinguish between the two explanations the paper is trying to adjudicate: that COVID-19 unmasks genuine, previously unrecognised hEDS, versus that COVID-era healthcare engagement surfaces previously uncoded symptom clusters that are then labeled hEDS. These are observationally equivalent in your dataset. Two things would be needed to separate them: a non-connective-tissue comparator such as a fibromyalgia or chronic-pain cohort, and chart-level validation of the hEDS coding, and you have candidly acknowledged that neither is feasible under your current N3C access. I accept that constraint as real, but its consequence is that the manuscript cannot lead with, or imply, the biological interpretation, because the surveillance/ascertainment interpretation is equally consistent with every result you present. Indeed, the encounter-adjusted rates in Figure 4, showing no change in diagnostic yield per encounter, arguably point toward the ascertainment reading and should be foregrounded rather than treated as a secondary observation.

The revision required is therefore primarily one of framing, and it must be thorough rather than partial:

1. The title must not imply that viral infection reveals or causes the disorder. Both the "unmask the disorder" phrasing and a bare "after COVID-19" construction carry that implication for a general readership. An honest associational framing centered on diagnosis timing (e.g., hEDS diagnosis being 'associated with' prior COVID-19 documentation) is what the evidence supports, and is what the reviewer and I are asking for.

2. Abstract, discussion, and conclusions must be reframed throughout as a descriptive study of comorbidity patterns, diagnostic timing, and healthcare utilisation in hEDS. Where the "unmasking" mechanism is raised, it should appear explicitly as one of at least two equally supported hypotheses, not as the favored reading with alternatives appended as caveats. The current text hedges in places but still leads with biology; the balance needs to shift so that the ascertainment explanation is given genuine parity.

3. Cohort comparability (Figure 3). Please address directly what the protective-direction odds ratios - death (0.43), pneumonia (0.66), positive PCR (0.69) - imply about the matched comparison. As one reviewer noted, differences of this magnitude suggest residual differences in baseline health, healthcare access, testing behavior, or surveillance intensity between cohorts, and this bears on how much interpretive weight the matched associations can carry. This should be discussed as a limitation on the comparison itself, not only as a treatment-pattern observation.

4. The summary statement that testing-related variables, pneumonia, and mortality showed no difference between groups is inconsistent with the revised Figure 3, where several of these do differ. Please correct this and any related passages so the narrative matches the current results.

To be clear, I am not asking for new analyses, given the N3C access limitations you have described. A focused, honest reframing that brings the title and interpretation into line with what the data can support would, in my view, put the manuscript in a strong position for acceptance.

With thanks for your continued work on this,

**Reviewers' Comments:** **Figure resubmission:**

**Reproducibility:**

To enhance the reproducibility of your results, we recommend that authors of applicable studies deposit laboratory protocols in protocols.io, where a protocol can be assigned its own identifier (DOI) such that it can be cited independently in the future. Additionally, PLOS ONE offers an option to publish peer-reviewed clinical study protocols. Read more information on sharing protocols at https://plos.org/protocols?utm_medium=editorial-email&utm_source=authorletters&utm_campaign=protocols

# Author comment: Author response to Decision Letter 3

**DOI:** 10.1371/journal.pdig.0001707.r006

30 Jul 2026

Attachment Submitted filename: Response To Reviewer Comments V4 (1).docx

# Editor report: Decision Letter 3

**DOI:** 10.1371/journal.pdig.0001707.r007

**Authors:** Laura Sbaffi

6 Aug 2026

Response to Reviewers Revised Manuscript with Track Changes Manuscript **Journal Requirements:**

1. Please provide separate figure files in .tif or .eps format.

For more information about figure files please see our guidelines:  https://journals.plos.org/digitalhealth/s/figures

https://journals.plos.org/digitalhealth/s/figures#loc-file-requirements

**Additional Editor Comments (if provided):** **1) The cohort size doesn't reconcile.**  Methods gives 7,786 + 24,031 - 5,709 overlap = 26,108, then removes 216 dual-coded patients and states the final cohort is 25,192. That subtraction gives 25,892. Meanwhile every column in Table 2 (sex, ethnicity, race) sums to **25,152** , and the Figure 6 and Supplementary Figure 1 legends also use 25,152, while Table 1, Table 2's header and the Figure 2 legend use 25,192 or 26,108. The Figure 2 legend actually calls 26,108 the *final* **2) Table 2's age column doesn't add up.** **3) Table 4 contains a practically impossible confidence interval.** **4) Thalassemia is reported both ways.** **5) Fix citations.** **Reviewers' Comments:** **Figure resubmission:**

**Reproducibility:**

To enhance the reproducibility of your results, we recommend that authors of applicable studies deposit laboratory protocols in protocols.io, where a protocol can be assigned its own identifier (DOI) such that it can be cited independently in the future. Additionally, PLOS ONE offers an option to publish peer-reviewed clinical study protocols. Read more information on sharing protocols at https://plos.org/protocols?utm_medium=editorial-email&utm_source=authorletters&utm_campaign=protocols

# Author comment: Author response to Decision Letter 4

**DOI:** 10.1371/journal.pdig.0001707.r008

7 Aug 2026

Attachment Submitted filename: Response to reviewers V5.docx

# Editor report: Decision Letter 4

**DOI:** 10.1371/journal.pdig.0001707.r009

**Authors:** Laura Sbaffi

17 Aug 2026

More Common Than We Thought: Hypermobile Ehlers-Danlos Syndrome, Comorbidity Burden, and Long COVID Risk in a National Cohort

PDIG-D-25-00524R4

Dear Mrs. Pearson,

We are pleased to inform you that your manuscript 'More Common Than We Thought: Hypermobile Ehlers-Danlos Syndrome, Comorbidity Burden, and Long COVID Risk in a National Cohort' has been provisionally accepted for publication in PLOS Digital Health.

Before your manuscript can be formally accepted you will need to complete some formatting changes, which you will receive in a follow-up email from a member of our team.

Please note that your manuscript will not be scheduled for publication until you have made the required changes, so a swift response is appreciated.

IMPORTANT: The editorial review process is now complete. PLOS will only permit corrections to spelling, formatting or significant scientific errors from this point onwards. Requests for major changes, or any which affect the scientific understanding of your work, will cause delays to the publication date of your manuscript.

If your institution or institutions have a press office, please notify them about your upcoming paper to help maximize its impact. If they'll be preparing press materials, please inform our press team as soon as possible -- no later than 48 hours after receiving the formal acceptance. Your manuscript will remain under strict press embargo until 2 pm Eastern Time on the date of publication. For more information, please contact digitalhealth@plos.org.

Thank you again for supporting Open Access publishing; we are looking forward to publishing your work in PLOS Digital Health.

Best regards,

Laura Sbaffi, PhD, MA, MSc

Section Editor

PLOS Digital Health

***********************************************************

**Additional Editor Comments (if provided):**

**Reviewer Comments (if any, and for reference):**
